# Advances and Challenges in SnTe‐Based Thermoelectrics

**DOI:** 10.1002/adma.202418280

**Published:** 2025-01-31

**Authors:** Lijun Wang, Raza Moshwan, Ningyi Yuan, Zhi‐Gang Chen, Xiao‐Lei Shi

**Affiliations:** ^1^ School of Chemistry and Physics ARC Research Hub in Zero‐emission Power Generation for Carbon Neutrality, and Centre for Materials Science Queensland University of Technology Brisbane Queensland 4000 Australia; ^2^ School of Material Science & Engineering National Experimental Demonstration Center for Materials Science and Engineering Jiangsu Province Cultivation base for State Key Laboratory of Photovoltaic Science and Technology Changzhou University Changzhou Jiangsu 213164 China

**Keywords:** device, material, SnTe, structure, thermoelectric

## Abstract

SnTe‐based thermoelectric materials have attracted significant attention for their exceptional performance in mid‐to‐high temperature ranges, positioning them as promising candidates for thermoelectric power generation. However, their efficiency is constrained by challenges related to electronic structure, defect chemistry, and phonon behavior. This review comprehensively summarizes advancements in SnTe thermoelectric materials and devices over the past five years, focusing on strategies to address these limitations. Key approaches include defect regulation, carrier transport optimization, and phonon engineering to enhance electrical conductivity, reduce thermal conductivity, and improve overall thermoelectric conversion efficiency. The review highlights breakthroughs in fabrication methods, doping and alloying, composite designs, and the development of novel nanostructures, with particular emphasis on 2D SnTe materials such as monolayers, bilayers, and thin films, which offer new opportunities for performance enhancement. Additionally, it provides an overview of SnTe‐based thermoelectric devices, covering fabrication techniques, performance optimization, stability, and flexible device development. Despite significant progress, challenges remain in developing n‐type SnTe materials, optimizing interfaces, ensuring long‐term stability, and maximizing conversion efficiency. This review fills gaps in the existing literature and offers valuable insights and guidance for future research aimed at improving thermoelectric properties, advancing device integration, and driving the commercial viability of SnTe‐based materials for practical applications.

## Graphical abstract

This article provides a comprehensive overview of SnTe‐based thermoelectric materials and devices, addressing key challenges in materials, devices, integration, and applications, while highlighting current progress, obstacles, and offering valuable insights for future advancements.

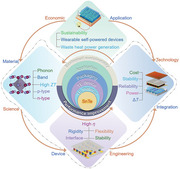

## 1 Introduction

In this era of major energy transition from “high‐carbon” to “low‐carbon”, the developments of low‐carbon, environmentally friendly clean energy and technologies for reutilizing wasted energy have received increasing attention. Statistics indicate that over 60% of the energy produced by fossil fuel combustion or nuclear power plants is lost as waste heat. If this underutilized waste heat can be effectively reclaimed to generate emission‐free renewable energy, it would yield significant economic and environmental benefits.^[^ ^1^ ^]^ Thermoelectric technology, capable of directly converting waste heat into electrical energy, plays a critical role in energy conversion. Thermoelectric systems are compact, highly adaptable, reliable, noise‐free, pollution‐free, and suitable for a wide range of applications. This green energy conversion technology operates using thermoelectric devices (TEDs) assembled from thermoelectric materials, enabling the transformation of waste heat into electricity.^[^ ^2^, ^3^, ^4^, ^5^, ^6^, ^7^ ^]^

Over the past two decades, thermoelectric technology has advanced rapidly, with its most promising applications including thermoelectric power generation (TEG)^[^ ^8^, ^9^ ^]^ and thermoelectric cooling (TEC).^[^ ^10^, ^11^ ^]^ TEC offers precise temperature control, flexible size design, diverse structures, and localized cooling effects, making it highly competitive in thermal management applications such as precision guidance, sensors, and 5G optical modules.^[^ ^10^ ^]^ In the field of TEG, thermoelectric technology holds even broader application potential.^[^ ^12^ ^]^ Examples include power supplies for remote data communication systems in oil and gas pipelines, generators for polar weather stations,^[^ ^3^ ^]^ radioisotope thermoelectric generators (RTGs) for deep space probes,^[^ ^13^ ^]^ and TEGs that convert automotive exhaust heat into electricity.^[^ ^14^ ^]^ With ongoing research, TEG technology has gradually evolved from solely waste heat recovery to integration with other energy technologies. For instance, TEG can be combined with solar photovoltaic systems, where the electricity generated by photovoltaics drives thermoelectric modules, further enhancing the energy conversion efficiency.^[^ ^15^, ^16^ ^]^ Integrating TEG with biomass energy and fuel cell technologies enables waste heat recovery during biomass combustion or fuel cell operation, achieving comprehensive energy utilization. This hybrid application not only optimizes the overall performance of the energy system but also enhances its economic and environmental viability.^[^ ^17^, ^18^ ^]^ Additionally, the combination of thermoelectric technology with other energy conversion methods such as piezoelectricity,^[^ ^19^, ^20^ ^]^ triboelectricity,^[^ ^21^ ^]^ and ferroelectricity,^[^ ^22^ ^]^ plays a crucial role in advancing the Internet of Things (IoT).^[^ ^23^ ^]^

The performance of TEG is primarily evaluated based on its thermoelectric conversion efficiency (*η*).^[^ ^24^ ^]^ This serves as a fundamental metric for assessing the capability of TEG to effectively convert heat energy into electrical energy. Under optimal conditions, the maximum conversion efficiency (*η*_max_) is given by:

(1) $$\eta_{\max}=\frac{\Delta T}{T_{h}}\frac{\sqrt{1+ZT_{\mathrm{av}g}}-1}{\sqrt{1+ZT_{\mathrm{avg}}}+T_{c}/T_{h}}$$

where *T*_h_ and *T*_c_ represent the temperatures at the hot and cold sides of the device, respectively, with Δ*T* = *T*_h_ – *T*_c_ denoting the temperature difference between the hot and cold sides. $\frac{\Delta T}{T_{h}}$ corresponds to the Carnot cycle efficiency, and $\frac{\sqrt{1+ZT_{\mathrm{av}g}}-1}{\sqrt{1+ZT_{\mathrm{avg}}}+T_{c}/T_{h}}$ is related to the material properties of the TEG. As shown in Equation **(1)**, *η* of a TEG is constrained by two primary factors: (1) the Carnot cycle efficiency, which can be improved by increasing the Δ*T* across the thermoelectric material; and (2) the thermoelectric figure of merit (*ZT*) of the selected material. A high *ZT* value indicates superior thermoelectric performance. *ZT* is a dimensionless parameter that integrates the Seebeck coefficient (*S*), electrical conductivity (*σ*), and thermal conductivity (*κ*) of the thermoelectric material, and expressed as:^[^ ^25^ ^]^

(2) $$ZT=\frac{S^{2}\sigma T}{\kappa }=\frac{S^{2}\sigma T}{\kappa_{e}+\kappa_{l}}$$

where *S* ^2^ *σ* is known as the power factor, primarily determined by the electrical transport properties of the material, while *κ* is influenced by both the electronic thermal conductivity (*κ*_e_) and lattice thermal conductivity (*κ*_l_), reflecting the thermal transport performance of the material.^[^ ^26^ ^]^ Since the 1950s, *ZT* has become an important parameter for evaluating the thermoelectric performance of materials. The issue of improving efficiency is linked to materials science, physics, and chemistry associated with the development of high *ZT* semiconductors. Researchers typically focus on the peak *ZT* (*ZT*_max_) and the average *ZT* (*ZT*_avg_) to enhance the *η*.

A review from 2008 pointed out that the goal of thermoelectric material research is to find bulk materials with a *ZT* value between 2 and 3 for both n‐type and p‐type materials (*η* of 15% to 20% for TEGs),^[^ ^27^ ^]^ while also minimizing parasitic losses (such as contact resistance, radiation effects, and interdiffusion with metals) and reducing manufacturing costs.^[^ ^28^ ^]^ To date, three material systems have achieved *ZT* values of 3.0: SnSe‐based thermoelectric materials reached a *ZT*_max_ of 3.1 at 783 K,^[^ ^29^ ^]^ Cu_2_Se‐based thermoelectric materials achieved a *ZT*_max_ of 3.0 at 1050 K, with a measured *η* of 13.4% under a Δ*T* of 518 K,^[^ ^30^ ^]^ and PbTe‐based thermoelectric materials achieved a *ZT*_max_ of 2.8 at 850 K, with TEGs made from this material reaching a *η* of 15.5% under a Δ*T* of 554 K.^[^ ^31^ ^]^ These materials are representative of mid‐temperature thermoelectric materials, with operating temperature ranges (523–973 K) that align with industrial waste heat temperatures.^[^ ^32^ ^]^ However, the *ZT* values of most mid‐temperature thermoelectric materials currently remain between 1 and 2.^[^ ^33^ ^]^ Therefore, developing high‐performance mid‐temperature thermoelectric materials remains an urgent task.

**Figure 1:**
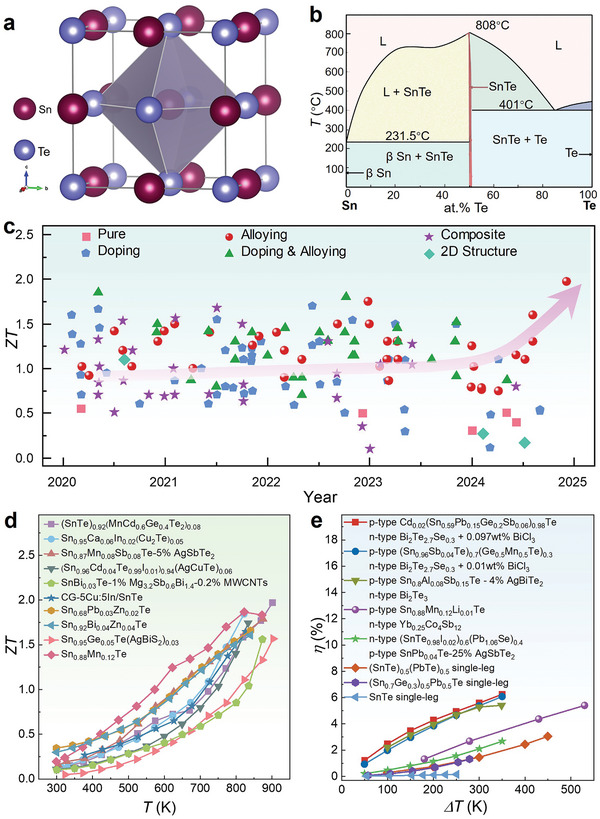
Exploring the characteristics and thermoelectric performance of SnTe‐based materials. a) Structural analysis of SnTe, showing Sn and Te atoms represented by large and small spheres, respectively. Copyright 2021, American Chemical Society. b) Phase diagram of the Sn‐Te system. c) Timeline of figure‐of‐merit (*ZT*) values for SnTe‐based materials over the past five years.^[^ ^39^, ^40^, ^41^, ^42^, ^43^, ^44^, ^45^, ^46^, ^47^, ^48^, ^49^, ^50^, ^51^, ^52^, ^53^, ^54^, ^55^, ^56^, ^57^, ^58^, ^59^, ^60^, ^61^, ^62^, ^63^, ^64^, ^65^, ^66^, ^67^, ^68^, ^69^, ^70^, ^71^, ^72^, ^73^, ^74^, ^75^, ^76^, ^77^, ^78^, ^79^, ^80^, ^81^, ^82^, ^83^, ^84^, ^85^, ^86^, ^87^, ^88^, ^89^, ^90^, ^91^, ^92^, ^93^, ^94^, ^95^, ^96^, ^97^, ^98^, ^99^, ^100^, ^101^, ^102^, ^103^, ^104^, ^105^, ^106^, ^107^, ^108^, ^109^, ^110^, ^111^, ^112^, ^113^, ^114^, ^115^, ^116^, ^117^, ^118^, ^119^, ^120^, ^121^, ^122^, ^123^, ^124^, ^125^, ^126^, ^127^, ^128^, ^129^, ^130^, ^131^, ^132^, ^133^, ^134^, ^135^, ^136^, ^137^, ^138^, ^139^, ^140^, ^141^, ^142^, ^143^, ^144^, ^145^, ^146^, ^147^, ^148^, ^149^, ^150^, ^151^, ^152^, ^153^, ^154^, ^155^, ^156^, ^157^, ^158^, ^159^, ^160^, ^161^, ^162^, ^163^, ^164^, ^165^, ^166^, ^167^, ^168^, ^169^, ^170^, ^171^, ^172^, ^173^, ^174^, ^175^, ^176^, ^177^, ^178^, ^179^, ^180^, ^181^, ^182^ ^]^ d) Temperature‐dependent *ZT* of typical bulk SnTe materials over the last five years.^[^ ^39^, ^48^, ^62^, ^64^, ^111^, ^123^, ^124^, ^141^, ^163^, ^165^ ^]^ e) Temperature difference (Δ*T*) dependence of thermoelectric conversion efficiency (*η*) in SnTe‐based thermoelectric devices.^[^ ^40^, ^84^, ^96^, ^107^, ^120^, ^160^, ^183^ ^]^

SnTe is considered a highly regarded mid‐temperature thermoelectric material, with significant advantages compared to other typical mid‐temperature thermoelectric materials such as PbTe and SnSe.^[^ ^34^ ^]^ SnTe exists in three crystal structures: rhombohedral (α‐SnTe), rock salt (β‐SnTe), and orthorhombic (γ‐SnTe). α‐SnTe is the low‐temperature phase, stable only below 100 K, while γ‐SnTe is the high‐pressure phase, which can be formed from β‐SnTe under high pressure.^[^ ^35^ ^]^ Typically, the form of SnTe used as a thermoelectric material is β‐SnTe, as shown in **Figure** **1**a. β‐SnTe has a cubic crystal structure, belonging to the $Fm\bar{3}m$ space group, with high symmetry and a lattice parameter of *a* = 6.3268 Å and *α* = 90°. In this structure, Sn and Te are located at the vertices and face centers of the cube, respectively, with each Sn atom surrounded by six Te atoms and vice versa, forming a tightly bonded ionic structure. Unlike PbTe, SnTe is more environmentally friendly. PbTe contains lead, which may pose environmental and health risks in practical applications, whereas SnTe is composed of Sn and Te, without toxic heavy metals, making it an eco‐friendlier choice that aligns with sustainable material design requirements. In contrast to the anisotropic SnSe, SnTe exhibits isotropy, meaning its physical properties are the same in all crystal directions. This simplifies the control and optimization of its thermoelectric performance, as there is no need to control crystal orientation in applications. Another significant advantage of SnTe is that it exhibits no obvious phase transitions within its operating temperature range. Figure 1b shows the phase diagram of the Sn‐Te binary alloy. SnTe is in a liquid phase at high temperatures and begins to solidify when the temperature drops below 808 °C, with a distinct eutectic reaction occurring at 401 °C. Due to the presence of the eutectic coupling band,^[^ ^36^ ^]^ eutectic microstructures can be obtained at the eutectic composition and its vicinity. It is noteworthy that SnTe alloys are not strictly stoichiometric compounds. The atomic percentage range of Te in the SnTe single‐phase region is (50.1% ± 0.1%) to (50.9% ± 0.1%),^[^ ^37^ ^]^ meaning the Te/Sn atomic ratio is slightly higher than 1, resulting in intrinsic high concentrations of cation vacancies in the material. Thermodynamically, SnTe exhibits stable single‐phase characteristics, which means that phase transitions have a negligible impact on its thermoelectric performance, thus enhancing its long‐term stability and performance across the operational temperature range.

In recent years, research on SnTe alloys has gradually increased, revealing the significant research potential of this system in the thermoelectric field. Despite this, the *ZT* value of pure SnTe (≈0.4) is significantly lower than that of PbTe, primarily due to its high cation vacancy concentration (room temperature *n* of 2 × 10^20^ to 1.5 × 10^21^ cm^−3^, where *n* is the hole carrier concentration), which results in lower *S*, higher *κ*_e_, and higher *κ*_l_, thus limiting its thermoelectric performance improvement. Inspired by the effective strategies used to enhance thermoelectric performance in PbTe, similar methods for controlling electronic and phonon transport have been applied to SnTe, leading to significant improvements in its thermoelectric properties.^[^ ^38^ ^]^ Figure 1c illustrates the evolution of the *ZT* values of SnTe‐based materials over the past five years, showing that Sn‐based thermoelectric materials modified by strategies such as doping and alloying have achieved higher *ZT* values, with the highest *ZT* reaching 1.97.^[^ ^39^ ^]^ Figure 1d summarizes the temperature‐dependent *ZT* values for the top 10 SnTe‐based thermoelectric materials with the best performance. **Table** **1** provides detailed thermoelectric performance data for SnTe‐based materials reported over the past five years. With further research into SnTe‐based thermoelectric materials, their potential continues to emerge, and the thermoelectric performance of these materials is gradually stabilizing, thus driving the development of TEDs based on SnTe alloys. Figure 1e summarizes the *η* values of single‐leg and double‐leg TEDs based on SnTe materials at different Δ*T*s. Jiang et al. developed a double‐leg TED based on p‐type Cd_0.02_(Sn_0.59_Pb_0.15_Ge_0.2_Sb_0.06_)_0.98_Te and n‐type Bi_2_Te_2.7_Se_0.3_ + 0.097wt% BiCl_3_, achieving a *η* of 6.3% at Δ*T* = 350 K,^[^ ^40^ ^]^ which is the highest *η* reported so far for SnTe alloy‐based TEDs. Although this *η* still lags behind the highest *η* value (15.5%) achieved by Pb‐based thermoelectrics,^[^ ^31^ ^]^ it is believed that with advances in device manufacturing technology and ongoing research, the *η* of SnTe‐based TEDs is expected to improve further.

**Table 1:** Summary of thermoelectric properties of SnTe‐based materials reported in five years (from 2020 to 2024). Here the units for *T*, *σ*, *S*, *S* ^2^ *σ*, *κ*, and *n* are K, S cm^−1^, µV K^−1^, µW cm^−1^ K^−2^, W m^−1^ K^−1^, and 10^20^ cm^−3^, respectively.

Material	Fabrication	Type	*ZT*_max_	*T* [K]	*σ* [S cm^−1^]	*S* [µV K^−1^]	*S* ^2^ *σ* [µW cm^−1^ K^−2^]	*Κ* [W m^−1^ K^−1^]	*N* [10^20^ cm^−3^]	Refs.	Year
Sn_3_MnTe_4_	FP+BTE	P	2.24	900	46.4	456	11			[185]	2022
(SnTe)_0.92_(MnCd_0.6_Ge_0.4_Te_2_)_0.08_	M + HPS	p	1.97	900	690.5	206	29.3	1.49	5.07	[39]	2024
Sn_0.95_Ca_0.06_In_0.02_(Cu_2_Te)_0.05_	M + SPS	P	1.85	823	1027	202.8	42.2	1.88	3.56	[124]	2020
Sn_0.87_Mn_0.08_Sb_0.08_Te‐5% AgSbTe_2_	M + SPS	p	1.8	873	886.9	178	28.1	1.36	4.8	[141]	2022
(Sn_0.96_Cd_0.04_Te_0.99_I_0.01_)_0.94_(AgCuTe)_0.06_	M + HPS	p	1.75	833	442	212	19.9	0.95	3.44	[111]	2022
Sn_0.88_Mn_0.12_Te	M + BM +A	P	1.7	873	335.6	237.3	18.9	0.97	3.85	[48]	2021
CG‐5Cu:5In/SnTe	CES + HPS	p	1.68	823	970.3	148.3	21.3	1.05		[165]	2021
Sn_0.68_Pb_0.03_Zn_0.02_Te	M + HPS	p	1.66	840	579.6	229	30.4	1.52		[64]	2020
Sn_0.71_Ge_0.2_Mn_0.07_In_0.02_Te	M+SPS	P	1.64	873	958.4	185	32.8	1.75	2.19	[186]	2024
Sn_0.92_Bi_0.04_Zn_0.04_Te	M + HPS	p	1.6	840	856.6	205	36	1.9	0.93	[62]	2020
Sn_0.95_Ge_0.05_Te(AgBiS_2_)_0.03_	M + BM + SPS	P	1.6	903	802.9	167.4	22.5	1.27		[123]	2024
SnBi_0.03_Te‐1%Mg_3.2_Sb_0.6_Bi_1.4_‐0.2% MWCNTs	M + BM + SPS	P	1.56	873	646	173.3	19.4	1.1	0.1	[163]	2021
Sn_0.92_Ag_0.03_Mg_0.08_Te	M	p	1.55	865	948.5	169	27	1.5	6	[70]	2021
Sn_0.94_Ag_0.03_Zn_0.03_Te	M + HPS	p	1.54	840	1108.3	197	41.7	2.27		[81]	2022
Sn_0.9_Te	M	p	1.53	750	2852.1	171.7	84.3	4.13	4.47	[158]	2020
(Sn_0.86_Cd_0.04_Sb_0.1_Te)_0.96_(Cu_2_Se)_0.04_	M + HPS	p	1.52	833	802	172	23.7	1.3	3.21	[150]	2023
Sn_0.96_Gd_0.04_Te	MA + SPS	p	1.5	900	1403.3	144.3	29.22	1.75	1.7	[56]	2023
Sn_0.88_Mn_0.12_Li_0.01_Te	M + SPS	p	1.5	873	399.8	233.5	21.8	1.27	2.25	[84]	2023
Cd_0.02_(Sn_0.59_Pb_0.15_Ge_0.2_Sb_0.06_)_0.98_Te	M + HPS	p	1.5	800	400	220	19.4	1.67	1.07	[40]	2022
SnTe‐15%MnTe‐2% Bi	M + HPS	p	1.5	850	400	221	19.5	1.1	0.3	[103]	2021
Sn_0.85_V_0.09_Sb_0.09_Te‐7% AgSbTe_2_	M + SPS	p	1.5	873	906.9	176.3	28.2	1.64	2.24	[112]	2022
Sn_0.92_Ge_0.04_Sb_0.04_Te‐5% Cu_2_Te	M + SPS	p	1.5	873	934.2	179.2	30	1.75	2.9	[126]	2020
SnAg_0.05_Te‐6% CdSe	M + A + SPS	p	1.5	873	574.1	180	18.6	1.1	1.43	[166]	2021
Sn_0.83_Ag_0.03_Mn_0.17_Te	M	p	1.45	865	922.4	164	24.8	1.48		[65]	2020
(Sn0_.98_In_0.01_Mn_0.01_Te)_0.75_(AgCuTe)_0.25_	M + SPS	p	1.45	800	589	175.9	18.2	1.1		[134]	2022
Sn_0.92_Ge_0.04_Bi_0.04_Te‐10% AgBiTe_2_	M + SPS	p	1.45	873	720	190.9	26.2	1.58	1.21	[145]	2023
(Sn_0.95_Cd_0.05_Te)_0.93_(Cu_2_Te)_0.07_‐1% I	M + SPS	p	1.42	823	746.4	192.3	27.6	1.6	3.1	[94]	2020
Sn_0.48_Cd_0.02_Ge_0.25_Pb_0.25_Te	M + SPS	p	1.42	873	443.4	230.7	23.6	1.5	0.59	[102]	2020
Sn_0.89_Mn_0.11_Te_0.99_(CuI)_0.01_	M + HPS	P	1.42	800	694.2	165	18.9	1.1		[128]	2021
Sn_0.9_Mn_0.12_Te	M + SPS	p	1.4	823	428.6	225	21.7	1.28		[91]	2021
(Sn_0.96_Sb_0.04_Te)_0.7_(Ge_0.5_Mn_0.5_Te)_0.3_	M + HPS	p	1.4	850	717.5	175.9	22.2	1.35	3.7	[107]	2022
Sb_2_Te_3_(Sn_0.996_Re_0.004_Te)_8_	M + A + SPS	p	1.4	773	864.1	182	28.62	1.58	16.7 (773 K)	[125]	2020
Sn_0.91_Mn_0.09_Te_0.99_I_0.01_	M + SPS	P	1.4	873	526.1	222.3	26	1.6	4.77	[132]	2021
(Sn_0.91_Mn_0.09_Te)_0.97_(AgBiTe_2_)_0.03_	M + HPS	p	1.4	823	491.3	193	18.3	1.07	5.69	[138]	2022
(Sn_0.88_Ge_0.12_Te)_0.97_(BiTe_1.5_)_0.03_	M + SPS	p	1.4	873	886.4	190	32	2		[140]	2022
Sn_0.85_In_0.05_Ag _0.10_Te	ST + SPS	p	1.38	823	1125.8	178	35.7	2.13	6.16	[61]	2020
Sn_1.03_Te‐5% MnSb_2_Se_4_	M + BM + SPS	p	1.36	800	866.6	153.8	20.5	1.25	5.78	[106]	2021
SnTe‐1.5% β‐Zn_4_Sb_3_	M + BM + SPS	p	1.32	873	899.4	171	26.3	1.74	0.79	[153]	2020
Sn_0.93_Mn_0.1_Te‐0.8at% BiBr_3_	M + SPS	p	1.31	873	590.3	201	23.85	1.59		[139]	2022
Sn_0.90_Mn_0.07_Bi_0.03_Te	M + HPS	p	1.3	850	471.5	208.5	20.5	1.34	1.94	[76]	2021
(SnTe)_0.86_(MnTe)_0.07_(Cu_2_Te)_0.07_	M + SPS	p	1.3	873	910.2	164.4	24.6	1.65	1.85	[79]	2022
Sn_0.98_V_0.02_Te	M + HPS	p	1.3	873	1061.6	187.7	37.4	2.51		[90]	2020
Sn_0.93_Mn_0.1_Te	M + A +SPS	p	1.3	873	444.3	229.5	23.4	1.59		[93]	2024
Sb_2_Te_3_(Sn_0.8_Ge_0.2_Te)_8_	M + SPS	p	1.3	723	778.5	179.2	25	1.39	7.57	[115]	2023
Sb_2_Te_3_(SnMn_0.08_Te)_10_	M + A +SPS	p	1.3	773	662.4	184.3	22.5	1.48	6.07	[117]	2023
Sn_0.94_Bi_0.04_Zn_0.02_Te‐5% Cu_2_Te	M + SPS	p	1.3	873	584.3	186.4	20.3	1.38	2.14	[130]	2021
Sn_0.94_Cd_0.06_Te‐0.08 Sb_2_Te_3_	M + A + SPS	p	1.3	753	655	187.6	23.1	1.3	2.13	[144]	2022
Sn_0.90_Bi_0.03_Mg_0.09_Te	M + SPS	p	1.3	873	631.4	187.4	22.2	1.49	3.48	[146]	2023
Sn_0.82_Mn_0.18_Bi_0.03_Te_0.91_I_0.09_	M + SPS	p	1.3	823	367.1	224.5	18.5	1.17	0.68	[148]	2023
(Sn_0.85_Sb_0.10_)_0.8_Ge_0.2_Te_0.8_Se_0.2_	M +A +SPS	P	1.3	823	740.7	180.0	24.0	4.67	0.66	[187]	2023
SnTe‐1.5% Bi_2_S_3_	ST + A + SPS	p	1.3	873	682	180	22.1	1.48	0.3	[171]	2022
Sn_0.72_Sb_0.16_Mn_0.12_Te	MS + HPS	p	1.27	773	566.5	193	21.1	1.28	4.48	[68]	2021
Sn_0.97_Y_0.03_Te‐5% Cu_2_Te	M + A + SPS	p	1.27	823	656	183.7	22.14	1.44	1.3	[177]	2023
3%Na+Sn_1.03_Te	M + SPS	p	1.26	898	1350	166.6	37.9	2.7	0.42	[55]	2022
(SnTe)_0.90_(CuSbTe_2_)_0.10_	M + SPS	p	1.26	823	994.8	155	23.9	1.56	4.37	[105]	2021
(SnTe)_2.91_(In_2_Te_3_)_0.03_(Ge_0.5_Mn_0.5_Te)_1.2_	M + A + HPS	p	1.25	850	705	181.8	23.3	1.58	12.46	[118]	2023
Sn_0.94_Bi_0.04_Te	M + SPS	p	1.23	873	956.7	184.2	32.46	2.3	4.3	[50]	2021
Sn_0.88_Sb_0.08_Mn_0.08_Te	M + HPS	p	1.23	873	632.7	184.6	21.6	1.53	4.49	[74]	2021
Sn_1.03_Te‐5% BiCuSeO	M + BM + SPS	p	1.21	835	471.9	179.3	15.2	1.05	0.32	[152]	2019
Sn_0.94_Ag_0.09_Y_0.05_Te	M + SPS	P	1.2	873	1157.8	146.5	24.9	1.81	2.27	[73]	2021
NaSn_10_SbTe_12_	M + SPS	p	1.2	873	890.7	177.3	28	2.1	2.5	[100]	2020
(SnGe_0.03_Te)_0.9_(Ag_0.5_Bi_0.5_Se)_0.1_ + 1.0 wt % ZnO	M + SPS	p	1.2	870	660.4	171.4	19.4	1.41	8.31	[108]	2022
(SnTe)_0.5_(PbTe)_0.5_	M + HPS	p	1.2	750	468.9	240	27.1	1.69	0.48	[160]	2020
(SnTe)_0.85_(NaSbSe_2_)_0.15_	CM + HPS	p	1.15	823	615.3	169.6	17.7	1.27		[121]	2024
Sn_0.88_Bi_0.08_Mn_0.08_Te	M + HPS	p	1.14	873	441.1	214.2	20.2	1.55	0.99	[74]	2021
(Sn_0.95_Mg_0.05_Te)_10_Sb_2_Te_3_	M + SPS	p	1.14	723	754.1	172.2	22.4	1.42	1.9	[133]	2021
Sn_0.99_In_0.01_Te‐(YbMg_2_Bi_2_)_0.03_	M + BM + SPS	p	1.14	823	362.6	230	19.18	1.38	0.87	[142]	2022
Sn_1.02_In_0.01_Te‐1% AgCuTe	M + BM + SPS	p	1.14	800	675.4	178	21.4	1.5	3.19	[143]	2022
Sn_0.88_Sb_0.08_Te	M + SPS	p	1.1	873	907.2	166	25	1.96	4.28	[51]	2021
Sn_0.92_Mn_0.11_Te	HPHT + A	P	1.1	775	630	182.6	21	1.48		[59]	2024
Sn_0.695_Ge_0.2_Bi_0.03_Sb_0.075_Te	M + HPS	p	1.1	873	899.2	162	23.6	1.87	2.19	[89]	2023
(Sn_0.70_Ge_0.15_Pb_0.15_)_0.86_Sb_0.04_Mn_0.1_Te	SHS‐HG‐SPS	p	1.1	873	694	157	17.2	1.37	3	[95]	2023
Sn_0.99_In_0.01_Li_0.125_Sb_0.125_Te_1.25_	M + HPS	p	1.1	873	844.8	165	23	1.82	5.75	[110]	2022
SnTe‐5% CuSbSe_2_	M + HPS	p	1.1	823	1021.9	152	23.6	1.77	3.28	[114]	2023
(Sn_0.98_Ge_0.05_Te)_0.91_(Sb_2_Pb_0.5_Te_3_)_0.09_	M + BM + SPS	p	1.1	778	632.5	172.4	18.8	1.35	2.1	[122]	2024
SnPb_0.04_Te‐12% AgSbTe_2_	M + SPS	p	1.1	823	1159.5	148.3	25.5	1.9		[131]	2021
Sn_0.66_Ge_0.3_Sb_0.04_Te	M + A + HPS	p	1.1	855	932.9	165	25.4	1.98		[147]	2023
SnTe nanosheets	AER	P	1.1	923	648.3	190	22.5	1.95		[147]	2020
Sn_0.94_In_0.02_Sr_0.04_Te	SPB‐C	p	1.09	823	1068.2	161.9	28	2.11		[82]	2023
SnTe‐Sb_0.06_	ST + SPS	p	1.08	873	802.8	170	23.2	1.88	1.39	[53]	2021
0.35% Cu/Sn_0.94_Mn_0.09_Te	M + CP + SPS	p	1.05	876	830.2	157.9	20.7	1.73		[176]	2023
SnTe‐7% Cu_1.5_Te	CM + A + SPS	p	1.04	823	1767.8	143.3	3.63	2.8		[178]	2023
SnTe + 5% AgBiSe_2_	M + A + HPS	p	1.02	860	720	175.2	22.1	1.86	2.58	[98]	2020
Sn_10_Sb_2_Te_13_	M + A + SPS	p	1.02	773	723.6	165	19.7	1.5	2.1	[101]	2020
Sn_1.02_In_0.01_Te‐5% Ag_2_S	M + BM +SPS	p	1.02	800	998	151	22.7	1.78	2.32	[113]	2023
SnTe‐8% MnSe	M + SPS	p	1.02	823	709.5	160.6	18.3	1.47	4.17	[119]	2023
Sn(Te_0.97_Se_0.03_)+0.03 Cu_1.75_Te	M + HPS	p	1.02	873	1349.6	126.8	21.7	1.85	16	[156]	2020
Sn_0.94_Ag_0.09_La_0.05_Te	M + SPS	p	1	873	939.8	161	24.36	1.87	1.81	[69]	2021
(Sn_0.99_Cd_0.03_Te)_0.97_(Cu_2_Se)_0.03_	M + HPS	p	1	800	587.9	189	21	1.68		[104]	2021
SnTe+6 wt.% WSe_2_	M + BM + HPS	p	1	873	751.5	152.6	17.5	1.53	1.33	[169]	2022
Sn_0.91_Sb_0.18_Te_1.18_	MS + SPS	p	0.95	823	740	183	24.2	2.21	2.96	[46]	2020
SnTe(Cu_2_Te)_0.06_‐1.5% ZnO	SSS + SPS	p	0.94	873	616	162.3	16.2	1.42	0.88	[173]	2022
Sn_0.89_Mn_0.08_Bi_0.03_Te	M + HPS	p	0.93	773	1020.6	154.3	24.3	2	2.13	[67]	2021
Sn_0.96_Bi_0.04_Te_0.98_Se_0.02_	M + HPS	P	0.92	849	643.8	188	22.75	2.1		[99]	2020
Sn_0.975_Sc_0.025_Te‐(Li_2_Te)_0.01_	M + A +HPS	p	0.91	873	458.8	201.1	18.6	1.78	0.69	[149]	2023
Sn_0.96_Cd_0.04_Te_0.88_Se_0.12_	M + A + SPS	p	0.9	873	512.8	188.1	18.1	1.76	1.96	[109]	2022
SnTe‐2% Bi_2_O_3_	M + SPS	p	0.9	823	838.8	164.6	22.7	2.08	0.92	[135]	2022
(Sn_0.6_Pb_0.4_)_0.995_In_0.005_Te	M + SPS	p	0.9	773	358	217.2	16.9	1.41	0.36	[136]	2022
(Sn_0.81_Sb_0.19_Te)_0.93_(AgCl)_0.07_	M + SPS	p	0.87	773	745	154	17.6	1.57	0.52	[127]	2021
Sn_0.96_Bi_0.04_Te‐5% CdSe	M + SPS	p	0.87	823	463.9	192	17.1	1.62	0.51	[151]	2024
Sn_0.73_Cu_0.12_Sb_0.15_Te	M + HPS	p	0.86	723	679.8	162	17.8	1.5	2.36	[77]	2022
Sn_0.685_Pb_0.285_In_0.015_Te_0.7_Se_0.3_	M + SPS	p	0.86	773	883	150.5	19.96	1.8		[88]	2023
(Sn_0.875_Pb_0.125_Te)_0.95_(AgSnSe_2_)_0.05_	M + SPS	p	0.86	873	1620	116.8	22.1	2.24	7.8	[116]	2023
(Sn_0.985_In_0.015_Te)_0.90_(AgCl)_0.10_	MST + SPS	p	0.86	823	1457	127.3	26.4	2.18	11.75	[159]	2020
Sn_0.61_Mn_0.09_Pb_0.3_Te_0.7_Se_0.3_	M + SPS	p	0.85	773	847.9	146.1	18.1	1.65		[87]	2022
(Sn_1.06_Te)_0.95_(InSb)_0.05_	M + BM + SPS	p	0.84	823	812.2	143.4	16.7	1.64	1.08	[155]	2020
(Sn_0.6_Ge_0.2_Pb_0.2_)_0.96_Sb_0.04_Te	SHS	p	0.82	873	437.5	184	14.8	1.58	0.14	[86]	2022
SnTe+1% PbS	ST + SPS	p	0.82	873	1084.5	135.8	20	2.13	6.18	[167]	2021
Sn_0.90_Bi_0.03_Sb_0.10_Te	M + A + HPS	p	0.8	823	730	169.2	20.9	2.16	2.61	[72]	2021
Sn_0.97_In_0.01_Cb_0.02_Te	SHS + PAS + HPS	p	0.8	888	597.7	176.4	18.6	2.07	2.28	[78]	2022
Sn_0.6_Pb_0.4_Te_0.98_I_0.02_	M + SPS	n	0.8	573	307.1	−221	15	1.1	0.11	[129]	2021
Sn_0.99_In_0.01_Te	M + HPS	p	0.8	873	932	157.1	23	2.54	0.38	[179]	2024
Sn_0.97_W_0.03_Te	M + A +SPS	p	0.79	823	1593	128	26.1	2.71	3.75	[92]	2023
SnTe_0.88_Cl_0.12_	M + SPS	P	0.78	873	812.3	157.3	20.1	2.3	0.74	[49]	2021
Sn_0.94_Bi_0.02_Cu_0.02_In_0.02_Te	M + HPS	p	0.78	823	922.2	153.8	20	2.18	0.97	[97]	2024
(Sn_0.7_Ge_0.3_)_0.5_Pb_0.5_Te	M	p	0.76	648	375.3	205	15.8	1.2	0.6	[96]	2024
Sn_1.03_Se_0.12_Te_0.87_Br_0.01_	M + SPS	p	0.75	823	792.3	156.6	19.43	2.13		[75]	2021
(SnTe_0.98_I_0.02_)_0.6_(Pb_1.06_Se)_0.4_	M + SPS	n	0.75	573	454.3	−167.2	12.7	1.02	0.4	[120]	2024
SnTeSb_0.05_	M + SPS	p	0.72	775	1334.4	133.4	23.8	2.56	0.66	[52]	2021
2% SnTe/Cu_3_SbSe_4_	M + BM + HPS	p	0.71	650	247.6	224.6	12.5	1.12	2.32	[164]	2021
Sn_0.96_Pb_0.01_In_0.03_Te	HT + SPS	P	0.7	873	1814.7	115	20.4	2.54	3.82	[63]	2020
Sn_0.88_Ti_0.03_Mn_0.09_Te	M + SPS	p	0.7	723	319.8	218	15.2	1.61		[71]	2021
Sn_0.89_Zr_0.02_Mn_0.09_Te	M + SPS	p	0.7	723	227.2	233.6	12.4	1.34	10.7	[71]	2021
Sn_0.6_Pb_0.4_Te_0.9875_Br_0.0125_	M + SPS	n	0.7	573	335.2	214	15.4	1.26	0.11	[137]	2022
SnTe‐1vol% SiC	M + HPS	p	0.7	823	1321.7	116.7	18	2.12	1.9	[154]	2020
(SnTe)_0.94_(Sb_2_Se_3_)_0.06_	ST + SPS	p	0.7	813	1041.4	128	16.6	1.87	11.4	[161]	2020
Sn_1.03_Te‐1vol% K_2_Ti_6_O_13_	OS‐SPS	p	0.7	873	669.5	155.8	16.3	1.91		[170]	2022
Sn‐90at%Te‐2at% Sb	M + SPS	p	0.69	633	520	165.3	14.2	1.31	0.77	[162]	2020
2% Ag‐plated SnTe	ST + CEP +SPS	p	0.67	823	1269	127.1	20.5	2.52	1.7	[172]	2022
SnTe+0.6wt% Ti_3_C_2_Tx MXene	MILD + ST + SPS	P	0.63	823	821.8	156	20	2.6	2.37	[168]	2021
Sn_0.94_La_0.06_Te	M + SPS	p	0.6	823	1539.5	111.1	19	2.61		[47]	2021
Sn_0.88_Zn_0.02_Sb_0.01_Te	M + HPS	P	0.6	773	723.2	151.2	16.7	2.13	1.34	[66]	2020
Sn_0.99_In_0.01_Te	M + HPS	p	0.59	710	1523.4	122.7	22.9	2.76	0.48	[54]	2022
SnTe	PM + SPS + SA	p	0.55	800	358.2	130.5	6.1	8.93	1.12	[41]	2020
Sn_0.94_Bi_0.06_Te	MST + SPS	p	0.54	823	1204.3	117.7	16.9	2.58	0.84	[57]	2023
Sn_1.01_Zr_0.02_Te	MST + SPS	p	0.54	673	472.5	181.5	15.6	1.94		[60]	2024
SnTi_0.03_Te	MST + SPS	p	0.52	723	854.7	141.3	17	2.37		[60]	2024
SnTe	M + HPHT	p	0.51	750	1061	118.9	15	2	1.06	[44]	2024
(SnTe)_0.95_(AgCl)_0.05_	MST + SPS	p	0.51	773	1970	103.1	20.8	3.16	8.9	[157]	2020
SnTe	SHS	p	0.5	873	644.2	139.3	12.5	2.1		[42]	2022
Sn_0.6_Pb_0.4_Te_0.94_Cl_0.06_	M + SPS	n	0.5	523	356.7	−193.7	13.4	1.4	0.07	[80]	2022
Sn_0.97_Zn_0.03_Te	MST + SPS	p	0.48	773	2006.7	90.7	16.5	2.75		[58]	2024
SnTe	M + HPS	p	0.4	773	1430	77	8.4	1.62	6.99	[45]	2024
Sn_0.95_Bi_0.05_Te+5wt% Graphene	MSS + HPS	p	0.35	523	4000	50	10	1.49	0.87	[174]	2022
SnTe	CPP + SPS	p	0.31	764	1580	91.1	13.1	3.24	2.60 (764 K)	[43]	2023
Sn_1.01_Ag_0.01_Cu_0.01_Te	M + SPS	p	0.29	773	886.4	95	8	2.13	1.87	[83]	2023
SnTe films	TDTR	P	0.27	600	2817.3	62.2	10.9	2.42		[180]	2024
2D SnTe	LPE	p	0.17	550	40	410.2	6.73	1.6		[182]	2024
Sn_0.89_Mn_0.09_Zn_0.02_Te	ST + SPS	p	0.11	473	686.5	66	11.68	1.23		[85]	2024
SnTe+1.5wt% SWCNTs	M + HPS	p	0.1	523	2914.9	54	8.5	4.45	4.12	[175]	2022
SnTe thin film	LPCVD	P		615	1364.2	78	8.3		35.1	[188]	2020
SnTe film	MBE	P		673	1544	107.3	17.3		2.27	[189]	2021
Sn_0.7_Sr_0.3_Te thin films	T.E + A	p		523	1134.2	161	29.4			[190]	2023
Sn_0.7_Sr_0.3_Te thin films	T.E + A	p		425	3322.7	108.2	38.9			[191]	2024
SnTe thin films	T.E	P		473	1.25	8033	81			[192]	2024

Abbreviations for materials: SWCNT is single‐walled carbon nanotubes; MWCNTs is multi‐walled carbon nanotubes; CG is coated grain; CG‐5Cu:5In is 5% of CuInTe_2_. CES is cation exchange of the surface; MILD is minimally intensive layer delamination method; HT is hydrothermal; ST is solvothermal; MST is microwave solvothermal; MSS is microwave solid‐state method; CP is chemical plating; CEP is chemical electroless plating; CM is colloidal method; SSS is solid‐state synthesis is; M is melting; MS is melt spinning; PM is powder metallurgy; SHS is self‐propagating high‐temperature synthesis; PAS is plasma activated sintering; self‐propagating high‐temperature synthesis under high‐gravity field combined with spark plasma sintering is abbreviated as SHS‐HG‐SPS; OS‐SPS is one‐step SPS; MA is mechanical alloying; SA is saturation annealing; BM is ball milling; A is annealing; HPS is hot‐pressing sintering; SPS is spark plasma sintering; HPHT is high‐pressure and high‐temperature; HP is high‐pressure; CPP is chemical precipitation process; LPCVD is low pressure chemical vapor deposition; T.E is thermal evaporation method; TDTR is time‐domain thermos‐reflectance; MBE is molecular beam epitaxy apparatus; AER is anion‐exchange reaction; LPE is liquid phase exfoliation; Single Parabolic Band model calculation is abbreviated as SPB‐C; first‐principles calculations and Boltzmann transport theory is abbreviated as FP+BTE.

Furthermore, with the continuous advancement of microelectronics technology, the demand for flexibility and miniaturization of emerging TEDs is steadily increasing. 2D single‐crystal semiconductors with layered crystal structures exhibit a certain degree of flexibility when reduced to thin layers. Their *σ* and carrier mobility (*µ*) are significantly higher in the in‐plane direction than in the out‐of‐plane direction.^[^ ^184^ ^]^ Biswas et al. found through first‐principles calculations and Boltzmann transport theory that 2D SnTe exhibits both thermodynamic and kinetic stability, showing excellent thermoelectric performance. Mn‐doped SnTe achieved a high theoretical *ZT* value of ≈2.24 at 900 K.^[^ ^185^ ^]^ Experimentally, Singh et al. synthesized 2D SnTe using flame melting and liquid‐phase exfoliation methods, achieving a *ZT* value of 0.17.^[^ ^182^ ^]^ These studies on nanostructured 2D SnTe thermoelectric materials may open up new opportunities for the development of ultrathin wearable self‐charging devices.

As mentioned above, SnTe‐based thermoelectric materials have made significant progress both theoretically and practically. In the future, SnTe‐based thermoelectric materials are expected to play a greater role in energy conversion, refrigeration technology, and green energy. To remain at the forefront of research, it is essential to closely monitor emerging trends in SnTe studies and systematically summarize key scientific advancements. This review examines the progress of SnTe‐based thermoelectric materials and devices over the past five years, with a focus on novel contributions and innovative breakthroughs. First, it analyzes the structural advantages of SnTe‐based thermoelectric materials, focusing on their basic characteristics. Next, strategies to enhance their thermoelectric performance are discussed, including fabrication methods, doping, alloying, and composite materials designs. An innovative highlight of this review is the comprehensive analysis of low‐dimensional SnTe materials, including monolayers, bilayers, and thin films, and their unique potential in thermoelectric device applications. Furthermore, this paper presents a novel summary of current research strategies and advancements in SnTe thermoelectric devices, emphasizing its application as an intermediate layer and underscoring its pivotal role in advancing the thermoelectric field. Finally, this review summarizes the challenges and bottlenecks encountered in SnTe‐based thermoelectric material research, especially the development of n‐type SnTe thermoelectric materials and devices, improving device efficiency and stability, and expanding the application scope of SnTe‐based thermoelectric devices. It offers valuable insights and a forward‐looking perspective for future research. By covering both bulk and low‐dimensional SnTe materials alongside their device applications, this review sets itself apart from previous studies, emphasizing the innovation and importance of these emerging advancements.

## 2 Fundamentals

Tin chalcogenide compounds (SnQ, Q = S, Se, Te) have garnered attention as thermoelectric materials due to their more environmentally friendly nature compared to PbTe. Among the SnQ series, only SnTe adopts the same rock salt structure as PbTe, making it an ideal candidate to replace PbTe. However, the thermoelectric performance of SnTe currently cannot match that of PbTe. There are two main reasons for this: First, the electronic structure of SnTe significantly influences its *S*. Although SnTe has the same valence band (VB) structure as PbTe, the energy difference (Δ*E*) between the two VB (light and heavy holes) in SnTe (Δ*E* ≈ 0.3–0.4 eV) ^[^ ^193^ ^]^ is much larger than in PbTe (Δ*E* ≈ 0.17 eV).^[^ ^194^ ^]^ This causes a reduced contribution of the heavy hole band to the *S*. Moreover, the small bandgap (*E*_g_ ≈ 0.18 eV at 300 K) between the VB and conduction band (CB) in SnTe leads to significant bipolar effects. Additionally, Sn vacancies result in an excessively high *n*, further reducing *S*. On the other hand, the microscopic structure of SnTe affects phonon scattering. The intrinsic vacancy defects in SnTe, combined with the fact that Sn has much smaller atomic mass than Pb, result in a much higher *κ*_l_ in SnTe compared to PbTe.^[^ ^195^ ^]^ Therefore, fine control of the electronic and phonon transport in SnTe is necessary to achieve performance comparable to PbTe. This section will overview the band structure, electronic structure, defect structure, and phonon structure of SnTe, aiming to provide theoretical foundations and experimental guidance for improving its thermoelectric performance.

### 2.1 Electronic Structure

**Figure 2:**
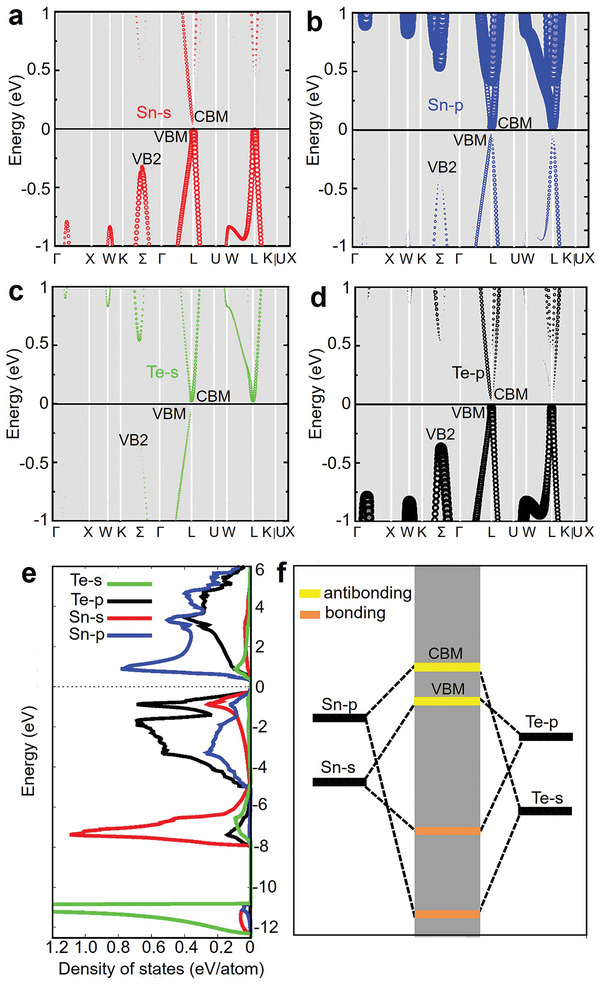
Band structures and interatomic interactions in pristine SnTe without spin‐orbit coupling. The orbital‐projected band structures of the pristine SnTe, excluding spin−orbit coupling (SOC), show the contributions from a) Sn‐s, b) Sn‐p, c) Te‐s, and d) Te‐p orbitals, represented by red, blue, green, and black dashed lines, respectively. VBM and VB2 denote the maximum valence band at the L point and the secondary maximum along the Σ‐line, respectively. e) Projected density of states (DOS) of SnTe without band inversion (no SOC). f) Diagram illustrating the interatomic interactions between Sn and Te atoms in the absence of band inversion. Reproduced with permission.^[^ ^197^ ^]^ Copyright 2021, American Chemical Society.

As mentioned above, the thermoelectric potential in SnTe as a thermoelectric material arises from its band structure.^[^ ^196^ ^]^ To perform band engineering, it is essential to understand the contribution of atomic energy levels to electronic bands. Zhang et al. used the Perdew−Burke−Ernzerhof generalized gradient approximation (GGA‐PBE) method to predict the electronic structure of SnTe, as shown in **Figure** **2**a–d.^[^ ^197^ ^]^ SnTe has a direct bandgap, with the valence band maximum (VBM) and conduction band minimum (CBM) both located at the L point. The VBM at the L point and the VB2 at the Σ point correspond to the light and heavy bands of SnTe, respectively, with the heavy band (Σ‐VB) having a higher effective mass (*m* ^*^).^[^ ^193^ ^]^ Figure 2a also shows that the contribution of the Sn‐s orbitals to the VBM is greater than that to VB2, meaning that by adjusting the interaction of the cationic s orbitals, the relative energy between the VBM and VB2 can be significantly altered, which is crucial for achieving band convergence.^[^ ^197^ ^]^ As is well known, in SnTe compounds, the s valence electron pairs (s^2^, known as lone pairs) are difficult to dissociate from the Sn atomic nucleus. Therefore, the Sn s orbitals contribute little to the CB. Additionally, the weak interatomic interactions reduce the energy gap between the bonding and antibonding states, effectively pushing up the bonding states and pulling down the antibonding states.^[^ ^198^, ^199^ ^]^ In the projected density of states (DOS) of SnTe (Figure 2e), the bonding states between Sn‐s and Te‐p orbitals are clearly observed in the range of −7.3 to −6.5 eV, while the antibonding states are found between −1.0 and −0.03 eV. Similarly, the bonding states between Sn‐p and Te‐s orbitals are in the range of −11.5 to −10.8 eV, and the antibonding states are between 0.03 and 0.9 eV. Based on these results, a schematic diagram of interatomic bonding behavior in SnTe is constructed (Figure 2f), indicating that the VBM primarily originates from the antibonding states between Sn‐s and Te‐p, while the CBM mainly comes from the antibonding states between Sn‐p and Te‐s. Therefore, by substituting Sn with cations in SnTe, the energy levels of individual atoms can be adjusted to tune the band structure, thereby altering the bandgap or achieving convergence of the VB.^[^ ^197^ ^]^

### 2.2 Defect Chemistry

In SnTe, spontaneously formed Sn vacancy (V_Sn_) defects significantly alter the *n*, *S* and *κ*, thus impacting its thermoelectric performance.^[^ ^61^ ^]^ Therefore, optimizing the concentration and distribution of V_Sn_ is a key strategy for improving the thermoelectric performance of SnTe materials.^[^ ^200^, ^201^ ^]^ Bipasha et al. combined *ZT* predictions with first‐principles simulations of intrinsic defects and *n* in SnTe to analyze the formation energies of cation vacancies (V_Sn_), anion vacancies (V_Te_), and anti‐site defects (Sn_Te_ and Te_Sn_). They found that under Sn‐rich and Sn‐poor growth conditions, V_Sn_ is the dominant acceptor defect with an extremely low formation energy, followed by the anti‐site defects Sn_Te_ and Te_Sn_. The only donor defect, V_Te_, has a relatively high formation energy across the entire stable region. The ultra‐low formation energy of V_Sn_ indicates its dominance in SnTe, this aligns with the observed degenerate p‐type behavior of SnTe in experiments. This also explains the difficulty in synthesizing stoichiometric SnTe and the inability to achieve n‐type performance due to the degenerate p‐type characteristics.^[^ ^202^ ^]^

**Figure 3:**
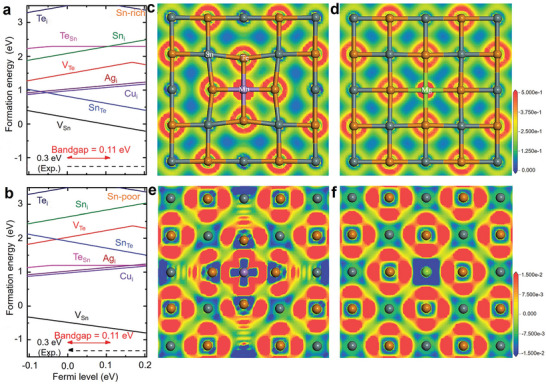
Defect formation energies and charge density in SnTe‐based materials. a,b) Calculated defect formation energies of SnTe as a function of the fermi level under Sn‐rich and Sn‐poor conditions. Reproduced with permission.^[^ ^203^ ^]^ Copyright 2021, Royal Society of Chemistry. c,d) Charge density maps and e,f) electron density difference maps of Mn‐ and Mg‐doped SnTe. Reproduced with permission.^[^ ^204^ ^]^ Copyright 2023, Elsevier.

Huo et al. investigated the formation energies of intrinsic and extrinsic defects (exogenous elements) in SnTe compounds through first‐principles calculations, as shown in **Figure** **3**a,b. The zero point represents the VBM of SnTe, and the slope indicates different charge states. The lower the formation energy, the easier it is for the defect to form under natural conditions. From Figure 3a,b, it can be observed that under Sn‐rich or Sn‐poor conditions, V_Sn_ in SnTe has a relatively low formation energy, indicating that the primary defect type is V_Sn_ acceptor defects, which cause SnTe to exhibit strong p‐type conductivity, especially under Sn‐poor conditions. The figures also show that doping with exogenous elements such as Cu_i_ and Ag_i_ can slightly increase the electron concentration in SnTe. However, since the formation energy of V_Sn_ is lower than that of interstitial defects Cu_i_ and Ag_i_, extrinsic doping only slightly enhances the n‐type conductivity of SnTe, highlighting the challenges in achieving n‐type doping.^[^ ^203^ ^]^

Li et al. calculated the defect formation energies for different concentrations of Mn and Mg atoms substituting Sn sites through first‐principles calculations (Figure 3c–f). Figure 3c,d shows that the electronic density provided by Mn doping is significantly higher than that of Mg and Sn atoms, and the electron density in the Mn─Te bond is also higher than in the Mg─Te and Sn─Te bonds. Additionally, the lattice distortion caused by Mn doping is much more pronounced than that caused by Mg doping. The Mn─Te bond length is much shorter than the conventional Sn─Te bond, and its polarity is significantly stronger than that of Sn‐Te and Mg─Te bonds. This results in a much higher defect formation energy when Mn atoms substitute Sn sites compared to Mg. The electron density difference maps in Figure 3e,f show that the shared electron pairs in the Sn─Te bond are almost equally distributed between Sn and Te, while in the Mn─Te bond, the electron pairs are more concentrated on the Te atom, indicating stronger polarity. Therefore, Te atoms cannot form shared electron pairs with Sn on the symmetric side of Mn, suggesting the distance between them is too large to form effective bonding. In contrast, Mg doping has a relatively smaller impact on the electron distribution.^[^ ^204^ ^]^ An in‐depth study of the defect chemistry of SnTe, particularly the formation energies of intrinsic and extrinsic defects, is essential for understanding its intrinsic conductivity type and explaining the mechanism by which doping elements affect carrier transport.

### 2.3 Phonon Behavior and Thermal Conductivity

One of the main reasons limiting the widespread application of SnTe is its high *κ*_l_. *κ*_l_ depends on phonon transport and is the only thermoelectric parameter that can be controlled independently, according to Drude's theory:^[^ ^205^ ^]^

(3) $$\kappa_{l}=\frac{1}{3}C_{V}\nu l=\frac{1}{3}C_{V}\nu^{2}\tau$$

To reduce *κ*_l_ and the overall *κ*, it is necessary to minimize the heat capacity (*C*_V_), phonon group velocity (*ν*), phonon mean free path (*l*), and relaxation time (*τ*). Since *C*_V_ is an inherent property of the material, it is difficult to reduce *κ*_l_ by modifying its value. In contrast, manipulating *ν* and *τ* is more feasible, as *ν* and *τ* are correlated through *l*. Minimizing *τ* involves enhancing phonon scattering through crystal defects or phonon‐phonon interactions, including point defects, dislocations, grain boundaries, nanostructures, and soft phonon modes. Simultaneously, reducing *ν* can be achieved through lattice softening. Over the past few decades, many methods have been proposed to reduce *κ*_l_. Among all strategies, nanostructuring has been proven to effectively reduce *κ*_l_. It should be noted that simply introducing nanoscale particles does not sufficiently reduce *κ*_l_, as phonon scattering corresponds to nanostructures with certain characteristic lengths, not all lengths. The characteristic length for phonon scattering should be approximately equal to or smaller than the phonon mean free path (MFP). In summary, when considering phonon scattering, emphasis is typically placed on achieving near‐full‐spectrum scattering to minimize the MFP of phonons, which often requires the coexistence of multiple scattering mechanisms. Therefore, distinguishing the contribution of each mechanism to the reduction of *κ*_l_ is challenging.

Slade et al. focused on the lattice softening mechanism in SnTe by preparing SnTe samples doped with nine different dopants and investigating the relationship between the *v* and *n*. Results showed that as the *n* of SnTe increased from ≈10^19^ to 10^21^ cm^−3^, *v* decreased approximately linearly, with reductions of 16% and 20%, respectively. This reduction was sufficient to lower *κ*_l_ by nearly 50%, indicating that *n* is the primary factor contributing to the softening of the lattice.^[^ ^206^ ^]^ Xie et al. measured the *v* of SnTe and GeTe/PbTe alloyed SnTe samples. The results indicated that GeTe/PbTe alloying significantly softened the lattice of SnTe. As the proportion of alloying agents increased, the decrease in *v* served as a clear indication of lattice softening.^[^ ^102^ ^]^

**Figure 4:**
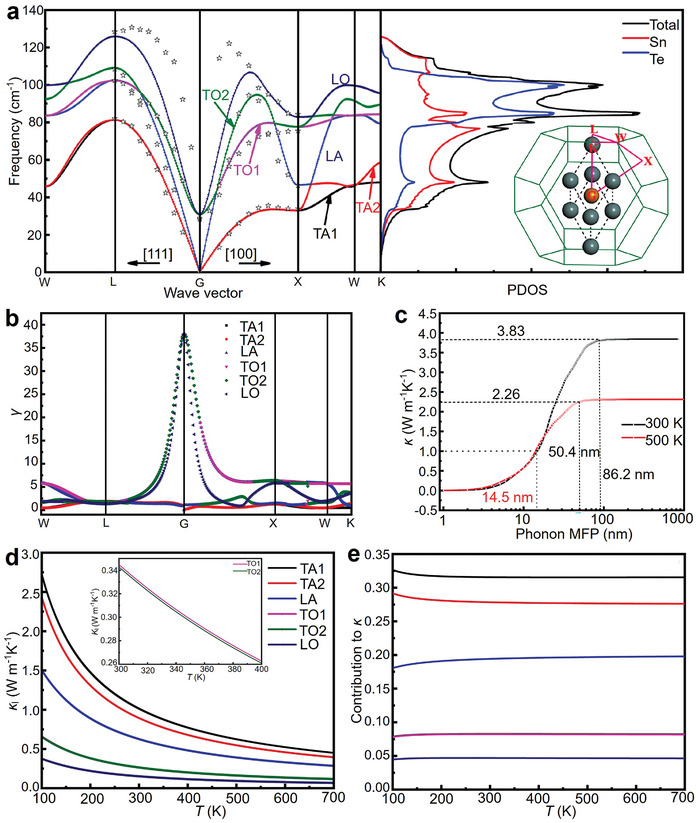
Contribution of phonon modes on lattice thermal conductivity. a) Computed phonon dispersion relations for SnTe across multiple high‐symmetry directions, along with the corresponding partial DOS (PDOS). b) Calculated anharmonic Grüneisen parameter (*γ*) across various high‐symmetry directions. c) Lattice thermal conductivity (*κ*_l_) as a function of phonon mean free path (MFP) at 300 and 500 K. d) *κ*_l_ for different polarizations as a function of temperature. e) Contributions of various phonon modes to the overall thermal conductivity (*κ*). Reproduced with permission.^[^ ^207^ ^]^ Copyright 2021, Elsevier.

Yang et al. systematically studied the quantitative contribution of each phonon mode to the overall *κ*_l_ in SnTe, discussing the importance of acoustic and optical modes as well as the impact of nanostructures with characteristic lengths on *κ*_l_.^[^ ^207^ ^]^ The results are shown in **Figure** **4**. Figure 4a presents the phonon dispersion relation of SnTe along high‐symmetry directions. Within the first Brillouin zone, there are six phonon modes, including two transverse acoustic branches (TA1, TA2), one longitudinal acoustic branch (LA), two transverse optical branches (TO1, TO2), and one longitudinal optical branch (LO). The optical modes are located in the high‐frequency range, and no bandgap between acoustic and optical modes is observed due to the small mass difference between Sn and Te. Figure 4b shows the total phonon DOS and partial phonon DOS (PDOS), revealing that Sn and Te atoms move together in the low and mid‐frequency ranges of the acoustic region, while the influence of Te atoms on the optical branches is relatively weak, especially in the frequency range above 110 cm^−1^. Figure 4c presents the Grüneisen parameter (*γ*) for SnTe, which shows that the *γ* value for optical modes is significantly higher than for acoustic modes. Larger *γ* values mean shorter phonon lifetimes, resulting in lower *κ*_l_ for the corresponding modes, explaining why optical phonons contribute less to *κ*_l_ than acoustic modes (see Figure 4d,e). Figure 4c also shows the dependence of total *κ*_l_ on the MFP at 300 and 500 K. The total *κ*_l_ increases with MFP until it stabilizes at 86.2 nm at 300 K or 50 nm at 500 K. When the MFP of phonons is smaller than 14.5 nm, *κ*_l_ drops to 1.0 W m^−1^ K^−1^ or lower. This suggests that nanostructures with characteristic lengths of 14.5 nm or smaller can reduce *κ*_l_ by at least 55.7% to 73.8% in the 300–500 K temperature range. Therefore, in addition to introducing various defects and alloying, designing nanostructures with characteristic lengths smaller than 14.5 nm is an effective strategy for significantly reducing *κ*_l_ in SnTe thermoelectric materials. Figure 4d shows that the contributions of the six phonon modes to *κ*_l_ decrease approximately linearly in the 300–700 K range. Figure 4e indicates that at 300 K, the acoustic modes contribute significantly more than the optical modes, with contributions of 78% and 22%, respectively. A systematic understanding of the physical processes behind phonon transport behavior can guide strategies to lower *κ*_l_ and improve thermoelectric performance.

## 3 Advances for Bulk Materials

In recent years, SnTe has attracted increasing attention. Many researchers have focused on modifying its unique dual‐VB electronic structure to improve electrical transport performance, while also slowing down phonon transport to optimize thermal transport properties. Key strategies include band engineering, carrier‐concentration optimization, synergistic engineering, and structural design. Although previous reviews have summarized these strategies,^[^ ^208^, ^209^ ^]^ significant new breakthroughs in thermoelectric performance of SnTe have been made over the past five years. Therefore, this section will introduce the latest research strategies related to SnTe, focusing on studies from the past five years, covering areas such as preparation, doping, alloying, composites, and structural design.

### 3.1 Fabrication Method

The synthesis process is a key step in obtaining the desired thermoelectric materials. SnTe‐based materials are typically prepared using traditional synthesis methods such as melting and solid‐state reactions. Additionally, high‐energy ball milling and mechanical alloying are also widely used to synthesize fine powders of SnTe. Recent studies have shown that SnTe can also be synthesized by self‐propagating high‐temperature synthesis. Chemical synthesis methods, such as the hydrothermal and/or solvothermal methods, are highly effective for producing fine powders with controlled morphology and size. Bulk forms of SnTe thermoelectric materials are usually prepared by combining two methods: one for synthesizing the powder, and the other for sintering the powder into bulk material. Sometimes to reduce the grain size, ingots made by melt casting are deliberately crushed before sintering.

#### 3.1.1 Melting

Melting technology has long been the traditional method for producing thermoelectric materials. Currently, the most commonly used method for preparing SnTe thermoelectric powders is still the meltin.^[^ ^65^, ^70^, ^96^, ^158^ ^]^ In laboratory settings, researchers tend to use the glass‐sealed melting method for directly producing small‐scale SnTe thermoelectric alloy ingots. For instance, Ahmad et al. improved the performance of SnTe by extruding excess Te to the grain boundaries during the solidification process of SnTe, thereby creating in situ Te nanoinclusions that enhance the thermoelectric properties.^[^ ^158^ ^]^ Pathak et al. used the melting method to prepare Ag and Mg co‐doped SnTe bulk materials, which achieved a density greater than 97%. The highest *ZT* value achieved was 1.55 at 865 K.^[^ ^70^ ^]^

Post‐processing steps are crucial for controlling the size and morphology of the SnTe material. Typically, SnTe thermoelectric alloys are prepared by the melting method, followed by crushing and grinding into fine SnTe grains. These fine grains are then sintered into dense bulk materials using sintering techniques such as spark plasma sintering (SPS) or hot‐pressing sintering (HPS). Thus, the combination of the melting method with SPS^[^ ^91^, ^112^, ^124^, ^141^ ^]^ or HPS^[^ ^39^, ^64^, ^103^, ^111^ ^]^ techniques remains one of the most common fabrication processes for SnTe thermoelectric bulk materials. For example, Liu et al. used the combination of melting and HPS to prepare (SnTe)_0.92_(MnCd_0.6_Ge_0.4_Te_2_)_0.08_, which achieved a *ZT* value of 1.97 at 900 K, marking the highest *ZT* record for SnTe to date.^[^ ^39^ ^]^ However, obtaining grain sizes larger than tens of micrometers through mechanical methods makes further densification of grain boundaries challenging. To refine the grain size, powders obtained from melting are typically ball‐milled to micron‐ and nanometer‐scale sizes before sintering.^[^ ^106^, ^123^, ^155^, ^163^, ^164^, ^169^, ^210^ ^]^

#### 3.1.2 Ball Milling

**Figure 5:**
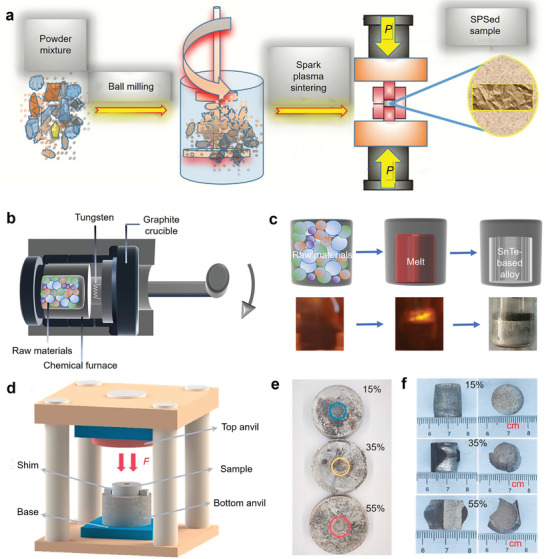
Fabrication of SnTe‐based thermoelectric materials. a) SnTe single‐phase alloy produced by mechanical alloying and spark plasma sintering (SPS). Reproduced with permission.^[^ ^214^ ^]^ Copyright 2023, Elsevier. b) Single‐phase SnTe‐based thermoelectric materials prepared by self‐propagating high‐temperature synthesis under high‐gravity field combined with SPS (SHS‐HG‐SPS) and c) different stages of the reaction. Reproduced with permission.^[^ ^95^ ^]^ Copyright 2023, Royal Society of Chemistry. d) Post‐processing SnTe based bulk materials by constrained hot compressing. Optical images of SnTe‐based samples at different compression ratios (15%, 35%, and 55%) after constrained hot compressing: e) with aluminum sleeve, f) without aluminum sleeve. Reproduced with permission.^[^ ^78^ ^]^ Copyright 2022, Elsevier.

Traditional melting methods require high temperatures and specific atmospheres, which are not only time‐consuming but can also result in the loss of elements through evaporation, leading to sample inhomogeneity. In contrast, ball milling has gained increasing attention in the thermoelectric field as a simpler process. The primary function of ball milling is to grind the powder obtained from melting, facilitating grain refinement. This process not only significantly reduces the particle size of the powder but also improves the material microstructure, which in turn enhances its mechanical and thermoelectric properties. Refining the grain size significantly facilitates the formation and optimization of grain boundaries, a crucial factor for enhancing the efficiency of subsequent sintering and processing steps.^[^ ^106^, ^113^, ^155^ ^]^ Ball milling includes both grinding and mechanical alloying. In addition to the grinding effect, the mechanical alloying process can synthesize compounds from elemental powders through mechanical‐chemical effects and in situ introduce nanostructures. Zhang et al. used the mechanical alloying method to prepare SnTe‐based nanocrystalline materials, which created many grain boundaries that significantly enhanced phonon scattering. Additionally, samples synthesized using both mechanical alloying and SPS processes exhibited unique microstructures with randomly distributed nanovoids, further suppressing the *κ*_l_. This combination of mechanical alloying and SPS effectively contributes to improving the thermoelectric performance of SnTe materials by controlling microstructure and phonon transport.^[^ ^56^ ^]^ Hakeem et al. prepared mechanically‐induced homogeneous SnTe solid solutions by ball milling Sn and Te elemental powders. The method achieved a batch yield of 100 g per cycle. The prepared powder samples were then sintered into dense bulk materials using SPS technology. The entire process is illustrated in **Figure** **5**a. This method provides a reference for bulk production of many other solid solutions in the same cycle, enabling scalable production while maintaining homogeneity in SnTe‐based thermoelectric materials.

#### 3.1.3 Self‐Propagating High‐Temperature Synthesis

Self‐propagating high‐temperature synthesis (SHS) is favored for its fast, one‐step process, low energy consumption, and strong scalability. It effectively maintains stoichiometry, making it suitable for the synthesis of various thermoelectric materials.^[^ ^211^, ^212^ ^]^ SHS is a process similar to a combustion wave, initiated by localized heating of the sample. In this process, the chemical reaction is confined to the self‐propagating combustion zone.^[^ ^213^ ^]^ Su et al. utilized an ultra‐fast SHS technique under a high‐gravity field to synthesize SnTe‐based thermoelectric materials. This method significantly reduced the synthesis time from several days to just a few seconds, enabling a much faster and more efficient production of SnTe materials while maintaining the desired properties.^[^ ^42^, ^86^ ^]^ Subsequently, they successfully synthesized a series of Ge, Pb, Sb, and Mn co‐doped SnTe‐based high‐entropy materials using the SHS‐HG‐SPS technique. Figure 5b briefly illustrates the combustion synthesis process, and Figure 5c shows the SnTe at different combustion stages of SHS‐HG. The SHS under high gravity effectively reduces the porosity of the samples. Meanwhile, the co‐doped SnTe increases the entropy due to lattice distortion and dislocation, thereby significantly lowering *κ*.

Zhang et al. successfully synthesized In and Cd co‐doped Sn thermoelectric materials combining resonant level and band convergence effects through SHS and plasma activated sintering (PAS) techniques.^[^ ^78^ ^]^ They then employed a constrained hot‐pressing technique (shown in Figure 5d) to obtain SnTe and Sn_0.97_In_0.01_Cd_0.02_Te bulk materials with different compression rates (0%, 15%, 35%, and 55%). Figure 5e,f shows the SnTe‐based samples and their corresponding optical images after constrained hot pressing. The V_Te_ formed during the constrained hot‐pressing process was arranged in a regular pattern, creating atomic‐ and nanoscale superstructure defects. These defects reduced the *n* in the compressed SnTe‐based materials, enhanced phonon scattering, and ultimately, the *ZT* value of the Sn_0.97_In_0.01_Cd_0.02_Te thermoelectric material with a 35% compression rate reached 0.8 at 888 K.

#### 3.1.4 Wet‐Chemical Synthesis

Wet chemical methods, also known as soft chemical methods, are synthesis techniques that involve chemical reactions in the liquid phase and are widely used in the preparation of thermoelectric materials.^[^ ^215^, ^216^ ^]^ Compared to traditional physical methods, wet chemical methods offer lower synthesis temperatures and better control over particle size, which facilitates the manipulation of material morphology and microstructure.^[^ ^53^, ^217^ ^]^ Currently, the most commonly used wet chemical methods include hydrothermal synthesis, solvothermal synthesis, and microwave‐assisted solution synthesis.^[^ ^159^, ^212^ ^]^ In recent years, wet chemical methods have also been applied to synthesize SnTe‐based thermoelectric materials. Lu et al. utilized a hydrothermal method to synthesize In and Pb co‐doped SnTe thermoelectric materials. Co‐doping resulted in grain refinement, induced ultra‐low *κ*, and enhanced the *S*, thereby improving thermoelectric performance and demonstrating the potential of the low‐energy‐consuming hydrothermal method.^[^ ^63^ ^]^

The solvothermal method is similar to the hydrothermal method, but its solvents or additives are typically organic compounds containing both hydrophobic and hydrophilic components, making the reaction conditions milder and more controllable.^[^ ^159^, ^161^, ^167^, ^218^ ^]^ Tian et al. synthesized SnTe powders decorated with Sb_2_Te_3_ nanosheets using a one‐step solvothermal method. After SPS sintering, an ion exchange reaction occurred between Sb_2_Te_3_ and the SnTe matrix, resulting in Sb doping and the formation of SnSb nanoparticles. This interface engineering enabled the SnTe−Sb_0.06_ sample to achieve a *ZT* value of ≈1.08 at 873 K.^[^ ^53^ ^]^ Moshwan et al. achieved In and Ag co‐doping through a simple solvothermal method, improving the electrical and thermal properties of SnTe‐based materials. The Sn_0.85_In_0.05_Ag_0.10_Te sample achieved a *ZT* value of ≈1.38 at 823 K.^[^ ^61^ ^]^

Recently, microwave‐assisted hydrothermal or solvothermal methods have emerged as promising synthesis techniques, attracting significant attention, especially in the research and development of thermoelectric nanostructures. Compared to conventional hydrothermal or solvothermal methods, microwave heating offers superior controllability, product morphology and size control, as well as chemical uniformity. In recent years, numerous studies have utilized microwave heating to synthesize thermoelectric nanostructures.^[^ ^57^, ^58^ ^]^ Wang et al. successfully synthesized (Sn_0.985_In_0.015_Te)_1−_ *_x_* (AgCl)*_x_* thermoelectric materials using a microwave solvothermal method in just 30 min. The process introduced a full‐scale hierarchical structure that significantly enhanced phonon scattering, reducing the *κ*_l_ of SnTe to 0.245 W m^−1^ K^−1^, surpassing the amorphous limit of *κ*_l_ for SnTe.^[^ ^159^ ^]^

#### 3.1.5 Melt Spinning

**Figure 6:**
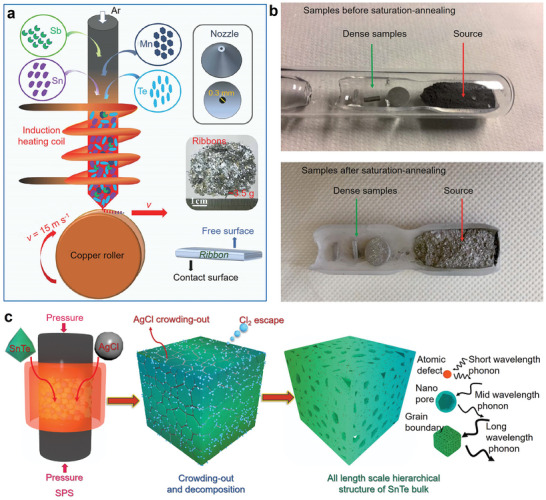
Preparation and processing techniques for SnTe materials. a) Schematic diagram of the melt spinning process for preparing SnTe and digital photographs of typical SnTe melt spun strips. Reproduced with permission.^[^ ^68^ ^]^ Copyright 2021, Elsevier. b) SnTe samples before saturation annealing and after saturation annealing. Reproduced with permission.^[^ ^41^ ^]^ Copyright 2020, Royal Society of Chemistry. c) Fabrication of all‐length‐scale SnTe pellets by SPS extrusion process. Reproduced with permission.^[^ ^157^ ^]^ Copyright 2020, Elsevier.

Melt spinning is a highly efficient method for rapidly cooling molten liquids. Due to its extremely fast cooling rate, it effectively suppresses grain growth, leading to the formation of nanostructures or even amorphous structures, which helps reduce *κ*_l_ and facilitates the achievement of high thermoelectric performance.^[^ ^219^ ^]^ For instance, Yang et al. utilized melt spinning combined with SPS to prepare Sb‐doped SnTe samples, optimizing both the electrical and thermal transport properties. The *ZT* value of the doped samples reached 0.95, which is 2.2 times higher than that of the pure SnTe phase.^[^ ^46^ ^]^ Yan et al. achieved a controlled synthesis of Sb‐ and Mn‐co‐doped Sn‐based thermoelectric materials by melt spinning, as illustrated in **Figure** **6**a. This approach introduced unique multiscale microstructures into Sn_0.84_Sb_0.16_Te, reducing its *κ*_l_ to as low as 0.55 W m^−1^ K^−1^ at 300 K. At 813 K, the material exhibited a *ZT* value of ≈1.27, representing a 110% improvement over pristine SnTe. Furthermore, Mn doping effectively tuned the electronic band structure, ultimately leading to a *ZT*_avg_ of up to 0.89.^[^ ^68^ ^]^

#### 3.1.6 Other Novel Approaches and Post‐Treatment

Nanostructuring has been proven to effectively enhance phonon scattering and reduce *κ*, leading to the emergence of novel methods for synthesizing nanostructured SnTe. For instance, 2D SnTe can be prepared by liquid phase exfoliation (LPE) methods;^[^ ^182^ ^]^ SnTe nanosheets can be synthesized using anion‐exchange reactions (AER);^[^ ^181^ ^]^ SnTe nanoparticles can be produced through colloidal methods (CM) ^[^ ^178^ ^]^ and chemical precipitation processes (CPP).^[^ ^43^ ^]^ High‐pressure and high‐temperature (HPHT) techniques have been used to induce nanoscale lattice distortions and dislocations in SnTe.^[^ ^44^, ^45^ ^]^ Additionally, thermal evaporation (TE),^[^ ^190^, ^191^, ^192^ ^]^ molecular beam epitaxy (MBE),^[^ ^189^ ^]^ and low‐pressure chemical vapor deposition (LPCVD) have been employed to fabricate SnTe thermoelectric thin films.^[^ ^188^ ^]^

However, nanostructures may negatively impact carrier transport, limiting the enhancement of thermoelectric performance. To maximize thermoelectric performance, post‐treatment processes for nanostructured materials have been developed and have shown promising results. To tackle this challenge, post‐treatment processes are utilized to reduce charge carrier scattering induced by nanostructures while preserving low *κ*. For isotropic SnTe particles, annealing treatments are commonly employed to improve uniformity, stability, and facilitate the formation of in situ nanostructures.^[^ ^171^, ^178^, ^190^ ^]^ After synthesizing or processing powders, an appropriate sintering process is always required to achieve densification, grain crystallization, further purification, and stabilization of the primary phase. In thermoelectric powder sintering, SPS and hot‐pressing are the most used techniques. In most cases, sintering into bulk material is the final step in manufacturing thermoelectric materials; however, post‐treatment is sometimes carried out depending on specific requirements. For instance, Ibrahim et al. utilized powder metallurgy to prepare SnTe particles, which were then sintered into bulk samples using SPS. The authors performed saturation annealing on SnTe powders and bulk samples under Sn‐rich conditions, as shown in Figure 6b. The results revealed that reducing the saturation annealing temperature from 973 to 823 K under Sn‐rich conditions effectively controlled the V_Sn_ concentration, thereby tuning the transport properties.^[^ ^41^ ^]^ Wang et al. utilized the extrusion effect during the SPS sintering process (Figure 6c) to induce multiscale defects within the SnTe matrix, significantly reducing the *κ*_l_.^[^ ^157^ ^]^

### 3.2 Doping

Doping is a crucial method for tailoring electronic structures and optimizing *n* to achieve potentially higher thermoelectric performance.^[^ ^186^ ^]^ It typically involves introducing a small amount of impurity atoms or defects into the host compound to modify its electrical and thermal properties. In thermoelectric materials, doping is employed to adjust the *n*, electronic structure, and lattice vibration characteristics of the host material, thereby achieving an optimized balance among *σ*, *S*, and *κ*.^[^ ^220^ ^]^ Since the impact of doping on the performance of thermoelectric materials is influenced by various factors, including the type of dopant, doping concentration, doping site, and the properties of the host material, its mechanism of action is quite complex. This subsection focuses on discussing the effects of single‐element doping, co‐doping, multi‐element doping, and compound doping on the thermoelectric performance of SnTe‐based materials over the past five years.

#### 3.2.1 Single‐Element Doping

To enhance the thermoelectric performance of SnTe, researchers have extensively explored approaches primarily focused on optimizing *n*, employing band engineering to increase the *S*, and enhancing phonon scattering to reduce *κ*_l_. These objectives can be achieved through single‐element doping. The presence of impurities and defects disrupts the periodic potential field created by the periodic arrangement of atoms, potentially introducing energy states within the bandgap that provide additional carriers. Thus, doping effectively modulates *n*. For example, Na‐doping,^[^ ^55^ ^]^ Sb‐doping,^[^ ^52^ ^]^ and Gd‐doping ^[^ ^56^ ^]^ have been shown to optimize the carrier concentration in SnTe, thereby tuning its electrical transport properties. Band convergence is a crucial strategy for enhancing the thermoelectric performance of SnTe. It refers to the scenario where the energy offset between two or more bands is sufficiently small, allowing carriers from multiple bands to contribute to electrical transport. In SnTe‐based thermoelectric materials, doping with elements such as Mn,^[^ ^91^ ^]^ Mo,^[^ ^221^ ^]^ W,^[^ ^92^ ^]^ and V ^[^ ^90^ ^]^ can effectively widen the bandgap and induce VB convergence, significantly boosting the *S*. Additionally, besides structural and compositional tuning to achieve band convergence, introducing non‐intrinsic energy levels (resonant levels) near the Fermi level can create peaks in the DOS, enhancing the *m* ^*^ and further increasing *S*. Elements such as Mo,^[^ ^221^ ^]^ La,^[^ ^47^ ^]^ W,^[^ ^222^ ^]^ In,^[^ ^54^ ^]^ and Bi^[^ ^50^, ^223^ ^]^ have been employed as resonant state dopants in SnTe thermoelectric materials. The structural defects introduced by doping also enhance phonon scattering, thereby reducing *κ*_l_. Particularly at high doping concentrations, the introduction of more point defects and impurity states significantly intensifies phonon scattering. For example, Zn‐doping,^[^ ^58^ ^]^ Cu‐doping,^[^ ^224^ ^]^ Mn‐alloying,^[^ ^48^, ^59^, ^91^, ^93^ ^]^ and Sb‐alloying^[^ ^52^ ^]^ have been shown to effectively lower *κ*_l_.

In addition to doping at the Sn cation sites, dopants can also be introduced at the Te anion sites. Band sharpening, as one of the key approaches for modifying the band structure, can significantly enhance material performance. Yang et al. achieved band sharpening in SnTe through Cl doping, successfully reducing the *m* ^*^ and significantly improving *µ*. This optimization enhanced *σ* while maintaining a stable *S*.^[^ ^49^ ^]^ The study revealed that the SnTe sample doped with 6% Cl achieved a maximum *S* ^2^ *σ* of ≈21 µW cm^−1^ K^−2^ at 873 K, representing a 30% improvement over pristine SnTe. Additionally, numerous micro‐ and nanoscale precipitates were observed in the Cl‐doped samples, significantly reducing the *κ*_l_ to ≈0.31 W m^−1^ K^−1^. At 873 K, the *ZT* value of the SnTe sample doped with 12% Cl increased from ≈0.4 (for pristine SnTe) to ≈0.78.

SnTe is the first experimentally confirmed topological crystalline insulator (TCI), exhibiting exceptional thermoelectric performance upon appropriate doping. However, it remains unclear whether doping preserves or disrupts its topological properties, thereby affecting its thermoelectric performance. To address this issue, Guo et al. experimentally reported a one‐to‐one correspondence between the thermoelectric properties and the topological phase transition of n‐type SnTe.^[^ ^137^ ^]^ n‐Type SnTe can achieve a topological phase transition only through Pb alloying, followed by Br doping to shift the Fermi level into the CB, thereby altering persistent p‐type conduction behavior. Ultimately, n‐type Sn_0.6_Pb_0.4_Te_0.9875_Br_0.012_ polycrystals and SnTe‐Pb‐Br crystals achieved peak *ZT* values of 0.7 and 0.8 at 573 K, with *ZT*_avg_ values of 0.42 and 0.58 over the temperature range of 300–823 K.

**Figure 7:**
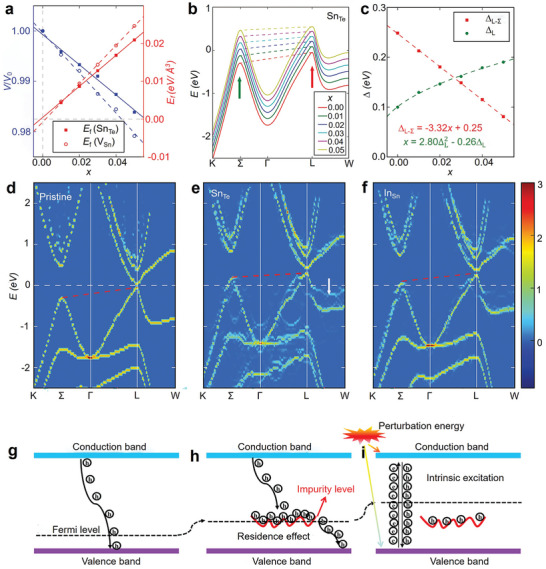
Band structure of SnTe with varying doping. a) Volume and formation energy of SnTe with varying V_Sn_ or Sn_Te_ Concentration. Here V_Sn_ denotes the Sn vacancy, and Sn_Te_ is the anti‐site defect (Sn substitute Te sites). b) Evolution of valence band in SnTe‐doped case. c) Direct bandgap at L (Δ_L_), and energy differences (Δ_L−Σ_) as functions of Sn_Te_ concentration (*x*). Band structure of d) pristine SnTe, e) Sn_Te_‐doped, and f) In_Sn_‐doped SnTe.^[^ ^200^ ^]^ Here In_Sn_ is the anti‐site defect (In substitute Sn sites). Reproduced with permission.^[^ ^200^ ^]^ Copyright 2020, Wiley. Schematic band structure of g) un‐doped SnTe at room temperature, h) impurity‐doped SnTe at low temperature, and i) impurity‐doped SnTe at high temperature. Reproduced with permission.^[^ ^50^ ^]^ Copyright 2020, Elsevier.

Muzaffar et al., using first‐principles calculations combined with Boltzmann transport theory, revealed the role of anti‐site defects (Sn_Te_) in enhancing the thermoelectric performance of SnTe while preserving its topological properties.^[^ ^200^ ^]^ They utilized the virtual crystal approximation (VCA) to investigate the effects of Sn_Te_ and V_Sn_ defects on the lattice volume and defect formation energy of SnTe, as shown in **Figure** **7**a. The results indicate that both types of defects lead to a reduction in cell volume, with V_Sn_ having a greater impact. When *x* exceeds 0.02 (where *x* is defined by Sn_1+_ *_x_* Te_1−_ *_x_* or Sn_1−_ *_x_* Te for Sn_Te_ and V_Sn_), the volume formation energy of Sn_Te_ is lower than that of V_Sn_. Given that the expected defect concentration is sufficiently high (Figure 7b) to effectively engineer the band structure, the study focuses on intrinsic defects within SnTe. Figure 7c shows that as the Sn_Te_ concentration increases, the topological bandgap at the L point (Δ_L_) widens, and the energy separation between the VBM at L and the sub‐VBM at Σ (Δ_L−Σ_) decreases, indicating significant bandgap broadening and band convergence. A comparison between the electronic structures without Sn_Te_ (Figure 7d) and with Sn_Te_ (Figure 7e) further confirms these effects, showing evidence of bandgap widening and band convergence, along with the emergence of resonance states near the Fermi level in Figure 7e. Compared to external dopants such as In_Sn_ (Figure 7f), Sn_Te_ exhibits stronger resonance state spectral weight and greater band convergence, as reflected by the slope of the red dashed line. Given the simultaneous presence of bandgap widening, band convergence, and resonance states induced by Sn_Te_, the resulting thermoelectric properties are expected to improve significantly. The thermoelectric performance of SnTe with anti‐site doping is predicted to surpass systems previously enhanced by external dopants. This finding highlights anti‐site doping as a promising and natural strategy for further optimizing the overall thermoelectric performance of SnTe and related systems.

As mentioned earlier, introducing resonance states in SnTe can optimize its thermoelectric performance, but effectively selecting suitable resonant dopants for SnTe requires a deeper understanding of the mechanism of resonance effects. Studies have shown that In‐doping can induce resonance effects in SnTe, significantly enhancing the *S* at room temperature. However, at temperatures above 600 K, the *S* of In‐doped samples becomes nearly identical to that of pure SnTe, indicating that the resonance effect diminishes at higher temperatures.^[^ ^225^ ^]^ Yang et al. proposed two triggering conditions for achieving resonance effects: first, the energy correlation between the impurity state and the host state; second, the positional correlation between the impurity state level and the Fermi level.^[^ ^50^ ^]^ Figure 7g presents the band structure model of SnTe at room temperature, where carriers transport between the CB and the VB, with the Fermi level positioned near the top of the VB. Electrical transport is typically governed by carriers near the Fermi level; therefore, the resonance state level determined by impurity states must be located near the Fermi level to influence the electrical transport properties. When the temperature is below 600 K, the perturbation energy is insufficient to provide carriers with enough energy, and their transport remains largely unaffected. However, impurity band states offer additional energy positions, as shown in Figure 7h. These positions enable the Fermi level to pin to the impurity states effectively. With the Fermi level and impurity energy levels well‐matched, the resonance state becomes activated, optimizing electrical transport performance. At temperatures above 600 K, the high perturbation energy is sufficient to excite carriers to shuttle between the VB and CB, as illustrated in Figure 7i. This movement shifts the Fermi level toward the CB, causing a mismatch between the impurity state level and the Fermi level, ultimately deactivating the resonance state.^[^ ^50^ ^]^

**Figure 8:**
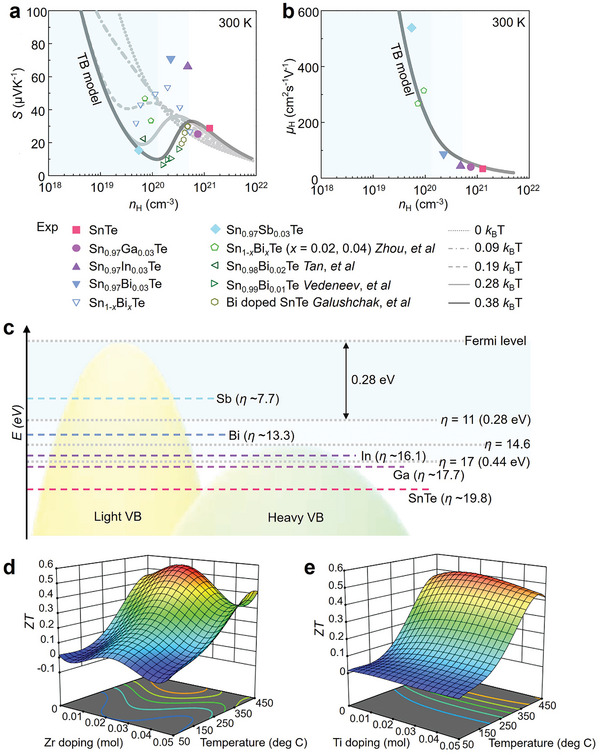
Effects of electron doping on the properties of SnTe. a) Seebeck coefficient (*S*) and b) carrier mobility (*µ*) of pristine and electron‐doped (Ga, In, Bi, and Sb) SnTe as a function of carrier concentration (*n*) from the experiments (symbols) and the two‐band (TB) model calculations (lines). Reproduced with permission.^[^ ^223^ ^]^ Copyright 2023, Wiley. c) The Fermi levels (dashed lines) of pristine SnTe and Sn_0.97_M_0.03_Te (M = Ga, In, Bi, and Sb) estimated by the TB model at 300 K.^[^ ^223^ ^]^ Copyright 2023, Wiley. 3D response surfaces and contour plots of *ZT* for d) Zr‐doped SnTe and e) Ti‐doped of SnTe. Reproduced with permission.^[^ ^60^ ^]^ Copyright 2024, Elsevier.

Precisely selecting and tuning doping elements is a critical strategy for enhancing the thermoelectric performance of SnTe. Park et al. utilized the TB (two‐band: one light valence band, LVB, and one heavy valence band, HVB) model to evaluate the effects of electronic dopants (Ga, In, Bi, and Sb) on SnTe.^[^ ^223^ ^]^ **Figure** **8**a shows the *n*‐dependent *S* of SnTe calculated using the TB model (depicted as the dark gray solid line), along with the *n*‐dependent *S* for Ga‐, In‐, Bi‐, and Sb‐doped SnTe samples and experimental values from the literature (represented as symbols). When *n* is below 1.3 × 10^20^ cm^−3^ (blue‐shaded region), the *S* calculated by the TB model gradually decreases. Within the *n* range of 1.3 × 10^20^ cm^−3^ to 6.2 × 10^20^ cm^−3^ (light‐blue‐shaded region), *S* suddenly increases and peaks ≈6.2 × 10^20^ cm^−3^, primarily due to contributions from the HVB. As *n* increases, the Fermi level at the LVB enters a region with significant contributions from the HVB. Figure 8a also indicates that the *n* values of Ga‐, In‐, Bi‐, and Sb‐doped SnTe samples are lower than that of pristine SnTe. The *n* for Ga‐doped samples lies in the HVB‐dominant region (non‐shaded), while In‐ and Bi‐doped samples span regions where both HVB and LVB contribute to electron transport (light blue‐shaded). For Sb‐doped samples, *n* falls into the LVB‐dominant region (blue‐shaded). The *S* values for pristine SnTe, Ga‐doped, and Sb‐doped samples align well with TB model predictions, while *S* values for In‐ and Bi‐doped samples show significant deviation from the TB model predictions. Even when LVB and HVB converge (light gray dashed line), the experimental data for Bi‐doped SnTe remain inconsistent with TB model results. This suggests that resonant states may form in Bi‐doped SnTe, similar to the behavior in In‐doped SnTe.

Figure 8b displays the *n*‐dependent *µ* for pristine and Ga‐, In‐, Bi‐, and Sb‐doped SnTe samples (represented as symbols), alongside TB model results (solid lines). No abrupt changes in *µ* are observed in the TB model. At high *n* values (>10^20^ cm^−3^), the presence of HVB mitigates the decline in *µ* with increasing *n*. The *µ* values for all samples, including In‐ and Bi‐doped SnTe, show reasonable agreement with the TB model. Since the *S* enhancement for In‐ and Bi‐doped SnTe exceeds TB model predictions (Figure 8a) without corresponding reductions in *µ*, and since the *S* enhancement for Bi‐doped SnTe cannot be explained by LVB and HVB convergence alone (light gray line in Figure 8a), it is likely that the high *S* in Bi‐doped SnTe arises from resonant states induced by doping. Figure 8c illustrates the band structure of pristine SnTe and the Fermi levels (shown as dashed lines) for Ga‐, In‐, Bi‐, and Sb‐doped samples as calculated by the TB model. The Fermi level is positioned near the peak of the LVB (highlighted in yellow), while the Δ*E* (shown in green) between the LVB peak and the HVB peak is 14.6 (0.38 eV at 300 K). As *n* increases, *S* decreases until Fermi level surpasses the HVB peak. Once Δ*E* between Fermi level and the HVB peak drops below 11, contributions from the HVB emerge, causing *S* to improve with increasing *n*. This trend continues until Fermi level falls below the HVB peak at 17. When Δ*E* between Fermi level and the HVB peak exceeds 17, LVB contributions become negligible, and *S* declines with further increases in *n*. For both In‐ and Bi‐doped samples, Fermi level resides in a region where both LVB and HVB contribute to transport. The formation of resonant states and optimized Fermi level positioning explain the high thermoelectric performance of In‐ and Bi‐doped SnTe at 300 K. These results highlight that using dopants that form resonant states, combined with placing the Fermi level near the LVB and close to the HVB peak, is a critical strategy for achieving high‐*ZT* SnTe.

The amount of dopant elements significantly impacts the thermoelectric performance of SnTe. Nasution et al. were the first to utilize mathematical modeling and machine learning methods, specifically the response surface methodology (RSM) and artificial neural network‐genetic algorithm (ANN‐GA), to predict the thermoelectric performance of SnTe‐based materials and determine the optimal dopant addition amount and operating temperature.^[^ ^60^ ^]^ The study examined 11 different concentrations of Ti‐ and Zr‐doped SnTe samples, with doping levels ranging from 0 to 0.05 m, and predicted thermoelectric performance over a temperature range of 50 to 450 °C. The results, shown in Figure 8d,e, indicate that the optimal Ti doping level (*x*) is ≈0.01, corresponding to the highest *ZT* value. For Zr doping, the peak *ZT* value lies within the *x* range of 0.02 to 0.03, with a maximum measured *ZT* value of 0.54 observed at 400 °C with the addition of 0.02 m Zr to SnTe. This study highlights the significant potential of RSM and ANN in determining optimal doping levels and operating temperatures.

**Figure 9:**
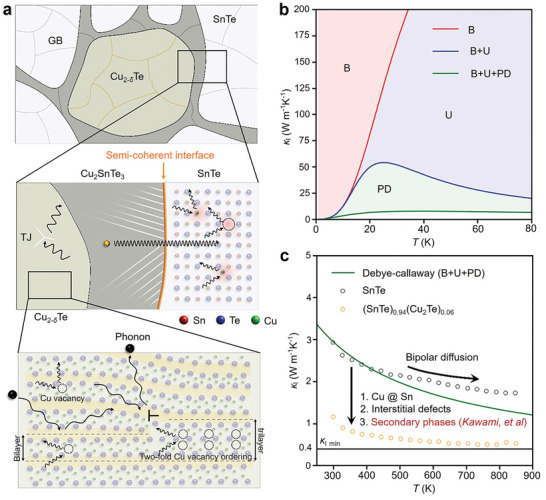
Phonon‐scattering mechanisms in Cu‐doped SnTe samples. a) Schematic representation of phonon‐scattering media from micro‐scale to atomic‐scale, with GB, TJ, and VdW indicating grain boundary, triple junction, and Van der Waals interactions. b) Calculated *κ*_l_ as a function of temperature (*T*) based on the Debye–Callaway model, accounting for boundary scattering (B), Umklapp process phonon–phonon scattering (U), and point defect scattering (PD). c) Calculated *κ*_l_ across typical thermoelectric operating temperatures for SnTe‐based compounds, alongside experimental *κ*_l_ data for SnTe and (SnTe)_0.94_(Cu_2_Te)_0.06_. Reproduced with permission.^[^ ^224^ ^]^ Copyright 2022, Wiley.

Light doping typically does not lead to significant second‐phase formation or phase separation but instead optimizes phonon scattering by introducing a small number of dopant atoms into the matrix. In certain cases, when the solubility of foreign atoms in the matrix is low, they may form nanoprecipitates^[^ ^226^ ^]^ or boundary composites.^[^ ^227^ ^]^ These foreign inclusions significantly influence phonon scattering behavior as their covered phonon wavelength range extends beyond what point defect scattering can affect, providing a diverse range of phonon scattering sources for different wavelengths of the spectrum. Moreover, these nanoprecipitates and composites may introduce other types of defects not present in the parent matrix, fostering the formation of a hierarchical phonon scattering architecture in thermoelectric materials, thereby contributing to improved thermoelectric performance.^[^ ^60^ ^]^ For example, Kawami et al. studied SnTe with Cu as a dopant and successfully achieved an ultra‐low *κ*_l_ close to the amorphous limit of SnTe.^[^ ^224^, ^228^ ^]^ The ultra‐low *κ*_l_ is primarily attributed to all‐scale phonon scattering introduced by Cu‐doping, covering a wide range of scattering mechanisms from microscale to atomic scale, as illustrated in **Figure** **9**a. Cu‐rich doping induces preferential precipitation of the Cu_2_SnTe_3_ secondary phase near the grain boundaries of the SnTe matrix, thereby forming a microscale secondary phase network. The semi‐coherent interface between Cu_2_SnTe_3_ and SnTe effectively reduces lattice mismatch, thereby facilitating thermoelectric transport. Furthermore, Cu‐rich doping introduces a metastable stoichiometric‐like Cu_2_Te secondary phase, which encompasses dislocation cores, ordered/disordered Cu vacancies (V_Cu_), dynamic grain boundary migration, and subsequent phase transformations during heating, resulting in various nanoscale and atomic‐scale defects. Additionally, Cu‐doping can lead to Cu substituting Sn sites or occupying interstitial positions within the SnTe lattice, forming atomic‐scale defects. Ultimately, these disordered Cu‐rich secondary phases, combined with point defects within the SnTe matrix, create additional phonon scattering centers, enabling the realization of ultra‐low *κ*_l_ in Cu‐doped SnTe. Figure 9b shows the effects of boundary scattering (B), Umklapp phonon‐phonon scattering (U), and point defect scattering (PD) on *κ*_l_ at low temperatures. The results indicate significant contributions from all three mechanisms, with boundary scattering being the dominant factor. Figure 9c compares experimentally measured *κ*_l_ with Debye‐Callaway model predictions over the 300–850 K range. For pure SnTe, *κ*_l_ aligns with the model predictions below 400 K but deviates significantly above 500 K due to bipolar diffusion. Compared to pure SnTe, Cu‐doped SnTe exhibits a pronounced reduction in *κ*_l_, attributed to point defects and the additional phonon scattering centers created by the disordered secondary phases introduced through Cu doping.

**Figure 10:**
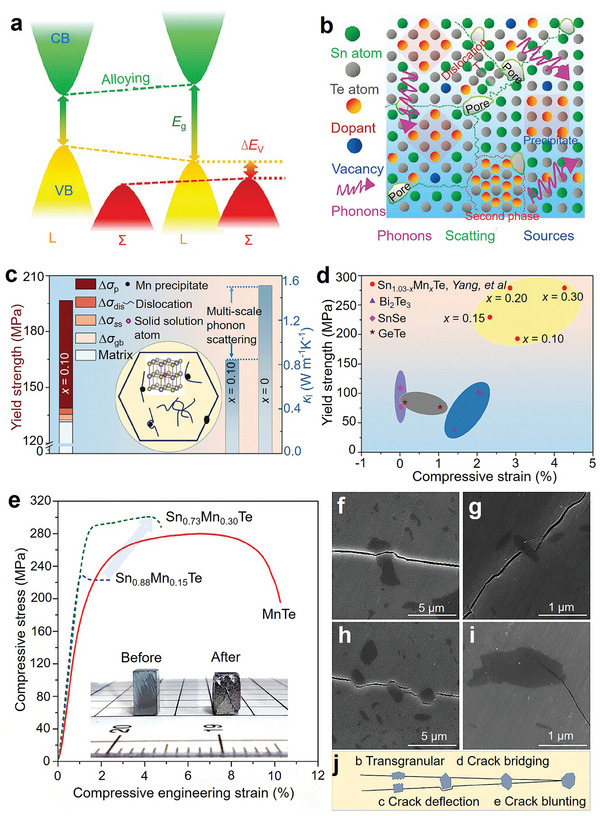
Band Structure, phonon scattering and mechanical properties in alloyed SnTe systems. a) Schematic of band structure engineering through alloying. b) Illustration of phonon scattering mechanisms from crystal imperfections in SnTe. Reproduced with permission.^[^ ^91^ ^]^ Copyright 2021, Wiley. c) Contribution of different strengthening mechanisms to yield strength of the Sn_0.93_Mn_0.10_Te sample and comparison of the *κ*_l_ between Sn_1.03_Te and Sn_0.93_Mn_0.10_Te sample, the inset shows the schematic diagram of the strengthening mechanisms in the Sn_0.93_Mn_0.10_Te sample, d) comparison of the room‐temperature compressive properties with some state‐of‐the‐art thermoelectric materials. e) Compressive stress‐strain curves for MnTe, with an inset optical image of MnTe samples before and after compressive testing. f–i) SEM images of Sn_0.83_Mn_0.20_ samples illustrating crack propagation. j) Diagrams depicting crack propagation. Reproduced with permission.^[^ ^93^ ^]^ Copyright 2024, Wiley.

In certain cases, foreign atoms exhibit high solubility in the matrix (i.e., high doping concentration), allowing them to be uniformly distributed and form a solid solution. This effectively adjusts the band structure and electronic transport properties through interactions between elements and the formation of new phases, thereby enhancing the *S* ^2^ *σ*.^[^ ^90^ ^]^ At the same time, additional defects and scattering centers are introduced, which further enhancing thermoelectric performance.^[^ ^90^, ^92^ ^]^ However, this may also impact the material stability.^[^ ^93^ ^]^ Mn has been identified as a doping element in various thermoelectric materials due to its high solubility and strong spin‐orbit coupling effects, making it a subject of extensive research. Mn doping significantly alters the band structure of SnTe, reducing the Δ*E* and increasing the bandgap.^[^ ^229^ ^]^ For example, Sun et al. demonstrated that Mn doping induces band convergence and an increased bandgap in SnTe (**Figure** **10**a) and revealed the hierarchical structure of Mn‐doped SnTe (Figure 10b).^[^ ^91^ ^]^ The study indicates that the band convergence induced by doping is closely related to the amount of dopant, leading to a higher *S*. Mn has a high solubility in SnTe, and when the Mn content reaches 8%, Mn‐rich nanoparticles are observed to aggregate at grain boundaries. When the Mn doping level increases to 10%, an α‐MnTe phase is detected precipitating between SnTe grains. As the Mn content increases, the grain size significantly decreases, indicating that multi‐scale precipitates and second phases may hinder grain boundary movement, resulting in grain refinement and enhanced phonon scattering.

In addition to optimizing the thermoelectric performance of SnTe, Mn doping‐induced structural defects also impact its mechanical properties. Yang et al. analyzed the mechanisms by which the extensive structural defects caused by Mn doping influence the mechanical properties of SnTe.^[^ ^93^ ^]^ Figure 10c illustrates the contributions of four structural strengthening mechanisms, including grain boundary strengthening (Δ*σ*_gb_), dislocation strengthening (Δ*σ*_dis_), solid solution strengthening (Δ*σ*_ss_), and precipitation strengthening (Δ*σ*_p_), to the yield strength of SnTe (Δ*σ*_Y_ = Δ*σ*_gb_ + Δ*σ*_dis_ + Δ*σ*_ss_ + Δ*σ*_p_). The study revealed that the compressive yield strength of the *x* = 0.10 sample increased by ≈60 MPa compared to the undoped SnTe sample, with precipitation strengthening being the primary mechanism, as contributions from grain boundaries, dislocations, and solid solutions were minimal. Furthermore, the hierarchical structure significantly reduced the *κ*_l_ of the *x* = 0.10 sample. Researchers compared the room‐temperature compressive behavior of Mn‐doped SnTe thermoelectric materials with some of the most advanced thermoelectric materials (Figure 10d). The results demonstrated that Mn‐doped SnTe samples exhibited excellent strength‐ductility performance. Figure 10e shows the compressive mechanical properties of MnTe bulk samples, where MnTe exhibited high strength and ductility. In SnTe samples with Mn content ranging from 0.15 to 0.30, the formation of MnTe resulted in improved yield strength. The primary mechanism for the increased yield strength was identified as dispersion strengthening. The crack propagation diagram in Figure 10j reveals transgranular cracking (Figure 10f), crack bridging (Figure 10g), crack deflection (Figure 10h), and crack blunting (Figure 10i) phenomena caused by MnTe precipitation. These findings indicate that the MnTe compound effectively disperses stress at the crack tip and dissipates crack propagation energy. Consequently, the observed increase in engineering strain in Mn‐doped SnTe samples is closely related to the formation of high strength‐ductility of MnTe. This combination of high strength and ductility provides localized sites for dislocation nucleation and accommodation, thereby enhancing plasticity.

#### 3.2.2 Co‐Doping

In SnTe, sometimes single‐element doping is insufficient to fully converge the VB, prompting the exploration of co‐doping strategies involving two or more elements to comprehensively optimize charge transport properties. Recent studies have also demonstrated that co‐doping in SnTe can further enhance the *S* and reduce *κ*_l_, thereby improving thermoelectric performance.^[^ ^63^, ^65^, ^66^, ^67^, ^69^, ^70^, ^72^, ^73^, ^74^, ^76^, ^79^, ^82^, ^83^, ^230^, ^231^, ^232^, ^233^, ^234^ ^]^ For instance, Pb and Zn co‐doped Sn‐based thermoelectric material Sn_0.68_Pb_0.3_Zn_0.02_Te achieved a *ZT* value of 1.66 at 840 K.^[^ ^64^ ^]^ This improvement is primarily attributed to the synergistic effect of Zn and Pb. Pb doping reduced the *κ* of SnTe, while Zn, acting as a versatile dopant, significantly enhanced *S* ^2^ *σ* by introducing resonant energy levels, increasing the bandgap, and strengthening the heavy‐hole VB at low temperatures. Bi and Zn co‐doping introduced resonant energy levels in SnTe, making the heavy‐hole VB dominant at room temperature, resulting in a record‐high *ZT* value of 0.3 at 300 K. Additionally, the formation of nanoscale precipitates significantly reduced *κ*_l_, achieving a peak *ZT* value of 1.6 at 840 K.^[^ ^62^ ^]^ Zn‐doping introduces resonant energy levels and induces ultra‐convergence, enhancing *S* at low temperatures. Meanwhile, Ag‐doping broadens the bandgap, promotes VB convergence, and prevents bipolar diffusion, thereby improving the performance at high temperatures. As a result, Sn_0.94_Ag_0.03_Zn_0.03_Te achieved a *ZT* value of ≈1.54 at 840 K.^[^ ^81^ ^]^ The resonant effect introduced by In‐doping, combined with the band convergence induced by Ag‐doping and the size effects achieved through the wet chemical method, enabled Sn_0.85_In_0.05_Ag_0.10_Te to reach a *ZT* value of 1.38 at 823 K.^[^ ^61^ ^]^ Cu/Mn co‐doped SnTe exhibited higher‐order anharmonicity, where the strongly anharmonic ionic potential reduced phonon lifetimes, ultimately contributing to the reduction of *κ*_l_.^[^ ^79^ ^]^

Muchtar et al. investigated the effects of co‐doping with transition metals Ti and Zr alongside Mn (Ti/Mn and Zr/Mn) on the structure, electronic properties, and thermoelectric performance of SnTe. They found that Ti/Mn and Zr/Mn co‐doping significantly improved the electrical transport properties of SnTe.^[^ ^71^ ^]^ This improvement is primarily attributed to Ti‐doping, which increased the DOS, and Zr‐doping, which optimized the electronic band structure by engineering carrier pockets and reducing the energy separation between light and heavy hole VB. Additionally, Mn co‐doping enhanced the interaction between carriers and magnetic moments. More importantly, Ti/Mn and Zr/Mn co‐doping softened the chemical bonds, reduced the anharmonic elastic modulus, increased internal strain, altered phonon group velocity, and induced phonon scattering, thereby significantly suppressing the *κ* of SnTe.

**Figure 11:**
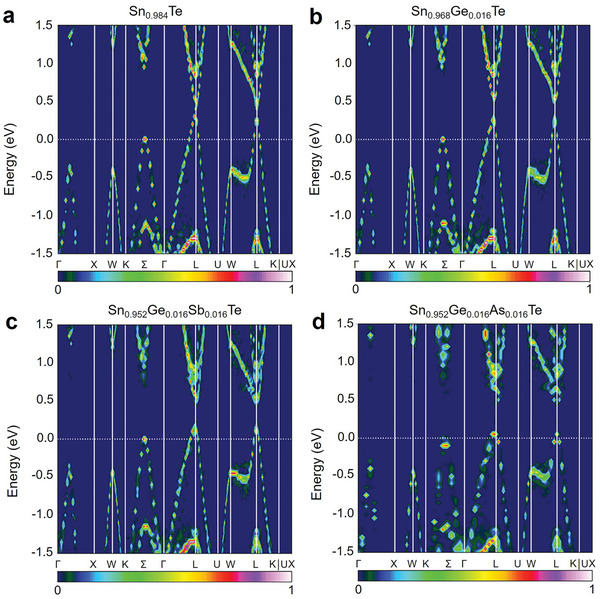
Band structure of SnTe with co‐doping. Unfolded band structures of a) Sn_0.984_Te, b) Sn_0.968_Ge_0.016_Te, c) Sn_0.952_Ge_0.016_Sb_0.016_Te, and d) Sn_0.952_Ge_0.016_As_0.016_Te. Reproduced with permission.^[^ ^197^ ^]^ Copyright 2021, American Chemical Society.

Zhang et al. investigated the band structures of SnTe with different co‐doping scenarios (Ge/Bi, Ge/Sb, and Ge/As) and found that introducing trivalent elements (Bi, Sb, and As) resulted in a rise in the Fermi level compared to the V_Sn_ system (Sn_0.984_Te, **Figure** **11**a). This shift was attributed to additional electrons introduced by the dopants compensating for the vacancies. Furthermore, the bandgap of Sn_0.968_Ge_0.016_Te slightly increased, as shown in Figure 11b, which helps suppress the high *n* in pristine SnTe. Co‐doping with Ge/Sb and Ge/As further increased the bandgap of SnTe from 0.06 to 0.16 and 0.20 eV, respectively (Figure 11c,d). The enlarged bandgap effectively mitigates bipolar diffusion. Since optimal thermoelectric materials require a large bandgap and a small Δ*E*, As or Sb co‐doped with Ge emerges as the most promising candidate for achieving superior electronic performance.^[^ ^197^ ^]^

#### 3.2.3 Multi‐Element Doping

Recent reports have highlighted new approaches involving the introduction of multiple dopants to simultaneously achieve various enhancements, including resonant state effects, bandgap widening, band convergence, and lattice softening.^[^ ^97^ ^]^ For instance, Xin et al. employed Pb, In, and Se co‐doping in SnTe to optimize bonding characteristics, V_Sn_, and resonant energy levels.^[^ ^88^ ^]^ Pb doping weakened the bonding strength, induced mass fluctuations, and stress distortion in the SnTe system, effectively reducing *κ*. Meanwhile, V_Sn_ site doping mitigated the *σ* loss caused by Pb, increased the bandgap, and elevated the resonant energy levels, leading to simultaneous improvements in *σ* and the *S*. As a result, the Sn_0.685_Pb_0.285_In_0.015_Te_0.7_Se_0.3_ sample achieved a *ZT* value of ≈0.86 at 773 K. Similarly, Kihoi et al. tri‐doped SnTe with Ge, Bi, and Sb to synergistically optimize its thermoelectric performance.^[^ ^89^ ^]^ Ge doping introduced amorphous (a‐Ge) precipitates, which contributed to reducing *κ*_l_. Bi and Sb co‐doping introduced dual resonant energy levels and distorted the DOS, increasing the *m* ^*^ of the band. This tri‐doped SnTe achieved a *ZT* value of ≈1.1 at 873 K.

### 3.3 Compound Alloying

An effective strategy for optimizing SnTe involves alloying it with compounds.^[^ ^118^, ^119^, ^235^ ^]^ In SnTe‐based thermoelectric materials, the high intrinsic vacancy concentration and high *κ*_l_ significantly affect thermoelectric performance. Therefore, defect chemistry plays a critical role in designing high‐performance SnTe thermoelectric materials.^[^ ^236^ ^]^ Recent studies have shown that alloying SnTe with I–V–VI_2_ compounds (such as AgSbTe_2_,^[^ ^237^, ^238^ ^]^ NaSbTe_2_,^[^ ^100^ ^]^ NaSbSe_2_,^[^ ^121^ ^]^ CuSbTe_2_,^[^ ^105^ ^]^ CuSbSe_2_,^[^ ^114^ ^]^ and AgSnSe_2_^[^ ^107^ ^]^) is an effective approach. This alloying can regulate vacancies in SnTe and enhance interactions between vacancy control and electro‐phonon transport properties by reducing *κ*_l_.

Alloying SnTe with AgBiSe_2_ significantly reduces *n*, resulting in moderate band convergence and an increase in the *m* ^*^. This improvement enhances the *S* over a wide temperature range. Additionally, the introduced point defects and nanoscale precipitates enhance phonon scattering, thereby reducing the *κ*_l_.^[^ ^98^ ^]^ Alloying SnTe with CuSbTe_2_ promotes VB convergence and increases the *n* in SnTe, effectively suppressing bipolar effects. Benefiting from the combined positive effects of VB convergence, increased vacancy concentration, and reduced *κ*_l_, the (SnTe)_0.90_(CuSbTe_2_)_0.10_ compound achieves a *ZT* value of 1.26 at 823 K.^[^ ^105^ ^]^ Similar to alloying with I–V–VI_2_ compounds, MnSb_2_Se_4_ also increases vacancy concentration, induces VB convergence, and introduces secondary phases of varied compositions. These effects contribute to the Sn_1.03_Te‐5%MnSb_2_Se_4_ sample achieving a relatively high *ZT* value of 1.36 at 800 K.^[^ ^239^ ^]^ In the SnTe‐3%CdTe system, alloying with 3% Cu_2_Se increases *n*, suppressing bipolar diffusion. The enhanced phonon scattering caused by mass and strain fluctuations due to alloying further reduces the *κ*_l_ of the SnTe‐CdTe system.^[^ ^104^ ^]^ Similarly, alloying with Sb_2_Te_3_ can simultaneously control vacancy concentration and *n*, leading to the formation of dense dislocations within the grains.^[^ ^101^ ^]^

**Figure 12:**
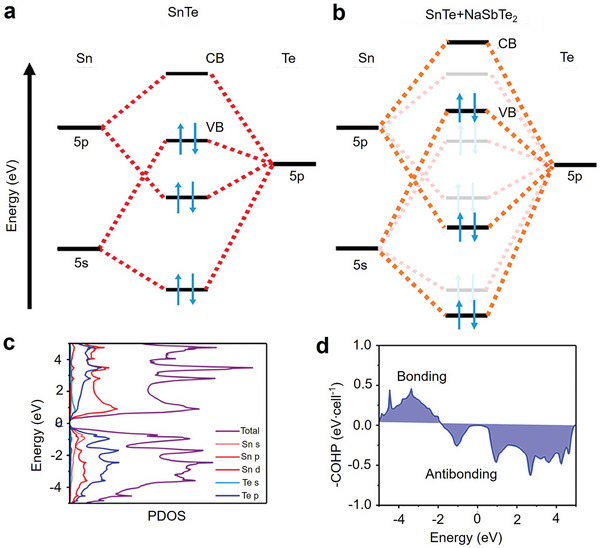
Effects of alloying on the structure and electronic properties of SnTe. a) Simplified molecular orbital (MO) diagram for a SnTe_6_ octahedron. b) Modified MO diagram reflecting lattice contraction induced by NaSbTe_2_. c) Calculated PDOS for pure SnTe. d) Crystal orbital Hamiltonian plot (COHP) for SnTe, indicating bonding (positive values) and antibonding (negative values) character. Reproduced with permission.^[^ ^100^ ^]^ Copyright 2020, American Chemical Society.

Slade et al. alloyed SnTe with NaSbTe_2_ and NaBiTe_2_, resulting in solid solutions of NaSn*_m_* SbTe*_m_*_+2_ and NaSn*_m_* BiTe*_m_*_+2_, respectively. Their results indicated that the introduction of NaSbTe_2_ and NaBiTe_2_ facilitated VB convergence while simultaneously narrowing the bandgap. However, the incorporation of NaSbTe_2_ nearly doubled the concentration of V_Sn_, primarily attributed to the antibonding characteristics of the VB in SnTe.^[^ ^100^ ^]^ **Figure** **12**a,b illustrates the molecular orbital (MO) diagram of the SnTe_6_ octahedron and modified MO diagram reflecting lattice contraction induced by NaSbTe_2_. In SnTe, the VB edge arises from the antibonding interaction between Sn‐5s and Te‐5p orbitals, induced by the Sn^2+^ 5s orbital. The PDOS calculated by density functional theory (DFT), shown in Figure 12c, confirms that Te‐5p and Sn‐5s orbitals contribute significantly to the total DOS. Concurrently, the crystal orbital Hamilton population (COHP) analysis of SnTe, depicted in Figure 12d, indicates that the VB edge predominantly exhibits antibonding characteristics. The antibonding nature of the VB in SnTe explains the formation of additional V_Sn_ when alloyed with NaSbTe_2_. The introduction of NaSbTe_2_, containing smaller Na^+^ and Sb^3+^ ions compared to Sn^2+^, compresses the lattice and applies “chemical pressure”, enhancing orbital overlap. This amplifies bonding and antibonding interactions, raising the energy of the VB edge. As shown in Figure 12d, the lattice compression destabilizes the antibonding electrons near the VB edge. The doubled vacancy concentration in SnTe upon NaSbTe_2_ alloying increases *n* and suppresses bipolar diffusion, thus improving the *S* ^2^ *σ*. Additionally, the incorporation of NaSbTe_2_ reduces *v*, endowing it with glass‐like *κ*_l_. Under the synergistic effects of band convergence, vacancy‐enhanced *n*, and lattice softening, NaSn*_m_* SbTe*_m_*_+2_ achieves a high *ZT* of ≈1.2 at 800–900 K.^[^ ^100^ ^]^

### 3.4 Combination of Doping and Compound Alloying

Building upon alloying, further doping can superimpose multiple effects, driving the exploration of synergistic alloying strategies to fully optimize charge transport properties.^[^ ^122^, ^127^, ^128^, ^130^, ^131^, ^132^, ^138^, ^139^, ^140^, ^143^, ^144^, ^145^, ^148^, ^149^, ^151^ ^]^ Xu et al. optimized the *n* and band structure of SnTe by alloying with AgBiS_2_, achieving a high *S* ^2^ *σ* of 22.5 µW m^−1^ K^−2^. Further doping with Ge introduced nanoscale precipitates and grain boundaries, reducing the *κ*_l_ to 0.3 W m^−1^ K^−1^ and resulting in a peak *ZT* of 1.6 at 903 K.^[^ ^123^ ^]^ Guo et al. found that alloying with LiSbTe_2_ and LiBiTe_2_ induced VB convergence in SnTe, while In‐doping introduced resonance levels. The alloying process introduced a high intensity of point defects and nanostructures, which significantly reduced fracture toughness, increasing the risk of failure due to external forces or thermal stress during processing or use. This highlights the need to balance mechanical properties while pursuing superior thermoelectric performance.^[^ ^110^ ^]^ Zhang et al. increased the solubility limit of Mn in the medium‐entropy SnTe‐GeMnTe_2_ system to ≈15%, resulting in near‐complete VB convergence and an increase in *m* ^*^. By finely tuning the *n* through Sb‐doping, significant improvements in *S* and *S* ^2^ *σ* were achieved. Ultimately, the medium‐entropy GeMnTe_2_ alloyed SnTe reached a peak *ZT* of 1.4 at 850 K.^[^ ^107^ ^]^ Xie et al. achieved band inversion in SnTe by alloying it with GeTe and PbTe, introducing multiple electronic valleys and significantly enhancing *S*. The high‐intensity dislocations introduced during alloying resulted in substantial lattice softening, thereby reducing *κ*_l_. Combined with Cd‐doping to widen the bandgap, the sample Sn_0.48_Cd_0.02_Ge_0.25_Pb_0.25_Te achieved a *ZT* value as high as 1.4 at 873 K.^[^ ^102^ ^]^ Chen et al. discovered that the highly converged transport VBs in SnTe‐MnTe alloys ensured excellent electronic performance. Introducing 2% Bi‐doping led to the formation of nanoscale particles and an intensive dislocation distribution, ultimately achieving a *ZT* of 1.5 at 850 K.^[^ ^104^ ^]^ Li et al. first introduced Ge into SnTe to increase its solubility, followed by alloying with Ag_0.5_Bi_0.5_Se and ZnO to optimize *n* and enhance *µ*. The addition of ZnO induced the precipitation of SnO_2_, which reduced *κ*_l_. The *ZT* value of (SnGe_0.03_Te)_0.9_(Ag_0.5_Bi_0.5_Se)_0.1_ + 1.0 wt.% ZnO reached 1.2 at 870 K.^[^ ^108^ ^]^

**Figure 13:**
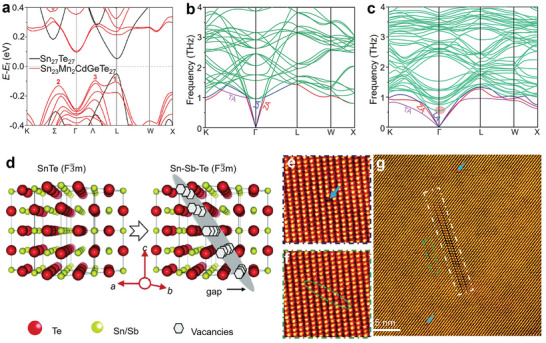
Effects of alloying on band structure and phonon transport properties of SnTe. a) Band structures of Sn_27_Te_27_ and Sn_23_Mn_2_CdGeTe_27_ with inclusion of spin–orbit coupling (SOC). The three‐valence band‐convergence related bands at L, Σ and Λ are labelled as 1, 2, and 3, respectively. Density functional theory (DFT) phonon dispersion of b) Sn_8_Te_8_ and c) Sn_6_MnCdTe_8_. Reproduced with permission.^[^ ^39^ ^]^ Copyright 2024, Royal Society of Chemistry. d) Predicted structure diagrams of Sn–Sb–Te. Aberration‐corrected scanning transmission electron microscopy (STEM) high‐angle annular dark field (HAADF) image of Sb_2_Te_3_(SnTe)_8_ sample showing e) a 1D V_Sn_ line and f) a 2D V_Sn_ gap. g) STEM‐HAADF image of Sb_2_Te_3_(Sn_0.996_Re_0.004_Te)_8_ sample featuring an extended gap‐like structure. Reproduced with permission.^[^ ^125^ ^]^ Copyright 2020, Royal Society of Chemistry.

Li et al. discovered that alloying ternary MnCdTe_2_ in SnTe could activate the lower‐energy Λ‐VB. Additionally, further Ge‐doping facilitated the alignment of the Λ‐VB with the L‐VB and Σ‐VB, resulting in triple‐band convergence (i.e., L, Σ, and Λ) as illustrated in **Figure** **13**a, significantly enhancing the *S* ^2^ *σ*. Moreover, Ge‐doping substantially improved the solubility of MnCdTe_2_ in the SnTe matrix, forming a hierarchical structure across all scales. Compared to the phonon dispersion of pristine SnTe (Figure 13b), the MnCdTe_2_‐alloyed SnTe exhibited overlap between low‐lying optical and acoustic phonon branches, enhancing optic‐acoustic phonon scattering and inducing stronger lattice anharmonicity, effectively shortening the phonon relaxation time. Simultaneously, the downward shift of optical modes reduced the Debye frequency (the maximum frequency of acoustic modes) from 1.45 to 1.35 THz (Figure 13b,c), flattening the acoustic branches and lowering the phonon group velocity, achieving ultralow *κ*_l_. Ultimately, the sample alloyed with 8% MnCdTe_2_ (containing 3.2% Ge) achieved a record‐breaking *ZT* of 1.97 at 900 K.^[^ ^39^ ^]^

Recent studies have shown that moderate interstitial defects can significantly enhance the thermoelectric performance of SnTe.^[^ ^117^, ^133^ ^]^ Xu et al. partially substituted Sn with Ge, enabling the regulation of local planar Sn defects in Sb_2_Te_3_(Sn_1‐_ *_x_* Ge*_x_* Te)_8_ samples. By increasing the density of interstitial‐like structures, the Sb_2_Te_3_(Sn_0.8_Ge_0.2_Te)_8_ sample achieved a *ZT* value of 1.3 at 723 K.^[^ ^115^ ^]^ Wang et al. fabricated Sn_1.03−2_ *_x_* V*_x_* Sb*_x_* Te samples, where the introduction of V interstitial atoms contributed to the formation of intensive dislocations in SnTe and reduced the Δ*E* between the light and heavy VBs. Upon alloying with AgSbTe_2_, the addition of Ag further decreased Δ*E*, promoted VB alignment, and adjusted the *n* to an optimal level. Consequently, the *ZT*_max_ reached 1.5, with a *ZT*_avg_ of 1.02.^[^ ^112^ ^]^ Xu et al. successfully engineered a bandgap structure in the SnTe system through alloying with Sb_2_Te_3_. In the selected Sb_2_Te_3_(SnTe)_8_ samples, van der Waals interstitial structures were introduced, as illustrated in Figure 13d. Aberration‐corrected scanning transmission electron microscopy (STEM) high‐angle annular dark field (HAADF) experiments confirmed the presence of these interstitial structures. The study revealed that high‐density cation vacancies formed linear defects along the [110] direction (marked by blue arrows in Figure 13e) and interstitial‐like defects (highlighted by green ellipses in Figure 13f). In Sb_2_Te_3_(SnTe)_8_ samples, cation vacancies tended to cluster along specific directions (Figure 13g), significantly reducing the *κ*_l_. Subsequently, Re‐doping was employed to precisely adjust the *n* at high temperatures, further enhancing the *S* ^2^ *σ*. As a result, a *ZT* value of 1.4 was achieved at 773 K for Sb_2_Te_3_(SnTe)_8_ samples doped with 0.4 at% Re, with a *ZT*_avg_ of 0.83 over the temperature range of 323 to 773 K.^[^ ^125^ ^]^

**Figure 14:**
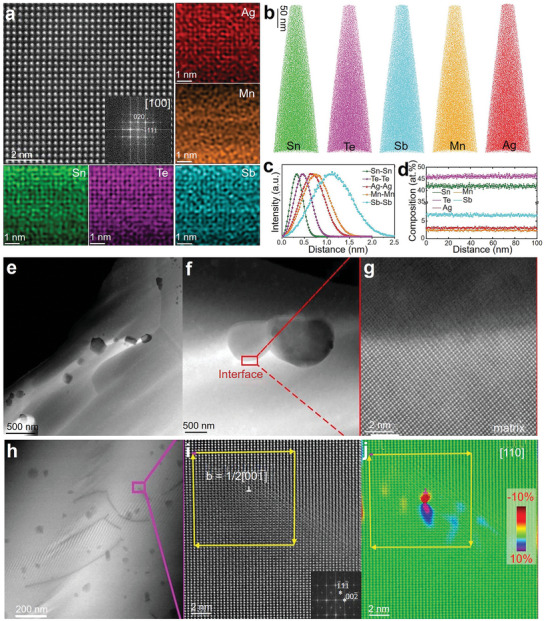
Characterization of Sn_0.87_Mn_0.08_Sb_0.08_Te–5% AgSbTe_2_ microstructures. a) Atomic‐resolution HAADF‐STEM image and energy dispersive X‐ray spectrometry (EDS): mappings of the SnTe matrix (inset fast Fourier transform (FFT) image). b) 3D reconstruction of elemental distribution. c) Nearest‐neighbor atomic distribution histograms for five elements. d) Composition profile from a 100 nm cuboid region along the vertical direction. e) Low‐magnification HAADF‐STEM images of precipitates. f) HAADF‐STEM image of a Mn‐rich nanoprecipitate. g) High‐magnification HAADF‐STEM image of the Mn‐rich precipitate‐SnTe matrix interface (bright contrast). h) Annular bright‐field (ABF)‐STEM images depicting dislocations (darker lines). i) Atomic‐resolution HAADF‐STEM image of a dislocation line's disappearing area (inset FFT image). j) Strain mapping corresponding to the HRTEM image by geometrical phase analysis (strain range indicated on the color bar: −10–10%). Reproduced with permission.^[^ ^141^ ^]^ Copyright 2022, Wiley.

Liu et al. alloyed SnTe with AgSbTe_2_ and doped it with Mn, and they discovered that the solubility limit of Ag in SnTe increased from 0.5% to over 7%. **Figure** **14**a clearly illustrates the positions of Ag, Sb, and Mn atoms within the Sn lattice. Figure 14b shows the 3D uniform distribution of Sn, Te, Sb, Mn, and Ag, indicating a high solubility of these dopants in SnTe. Figure 14c,d depicts the nearest neighbor atomic distribution histograms and vertical compositional distribution of the five elements, respectively. Figure 14e reveals that the average size of nanoscale precipitates in the matrix is ≈10 nm, with a magnified HAADF‐STEM image (Figure 14f) clearly displaying the precipitates. Figure 14g shows incoherent interfaces between Mn‐rich precipitates and the SnTe matrix due to significant lattice mismatches. Figure 14h presents a typical dislocation array, and Figure 14i,j further detail the atomic arrangement around the dislocation cores. These dislocations and nanoscale precipitates serve as additional phonon scattering centers, significantly reducing *κ*_l_. Consequently, Sn_0.87_Mn_0.08_Sb_0.08_Te‐5%AgSbTe_2_ achieved a peak *ZT* value of 1.8 at 873 K.^[^ ^141^ ^]^

(Cu, Ag)_2_X (X = S, Se, or Te) compounds,^[^ ^240^ ^]^ characterized by their extremely low *κ*_l_, are considered “phonon liquid, electron crystal” (PLEC) materials and have been employed to suppress the *κ*_l_ of SnTe.^[^ ^134^, ^150^ ^]^ For example, Hussain et al. embedded Cu_2_Te nanostructures into SnTe using a phase separation strategy, significantly reducing the *κ*_l_ of SnTe. Additionally, co‐doping with Ca and In effectively widened the bandgap, thereby suppressing bipolar thermal conductivity (*κ*_b_). The synergy of these electronic and thermal effects resulted in a *ZT* value of ≈1.85 at 823 K.^[^ ^124^ ^]^ Li et al. demonstrated that low‐content Ge and Sb co‐doping induces VB convergence in SnTe, significantly enhancing the *S*. Through a phase separation strategy, they successfully in situ introduced Cu_2_Te nanostructures into Ge and Sb co‐doped SnTe, significantly reducing *κ*_l_. As a result, a *ZT* value of 1.5 was achieved at 873 K in Sn_0.92_Ge_0.04_Sb_0.04_Te‐5% Cu_2_Te.^[^ ^126^ ^]^ Wu et al. alloyed the PLEC material AgCuTe into SnTe, aiming to reduce *κ*_l_ by introducing multiscale defects. Additionally, Cd‐doping was employed to promote VB convergence, thereby enhancing the *S* of SnTe. Further incorporation of iodine (I) was used to fine‐tune the *n* in AgCuTe‐Cd co‐doped SnTe. As a result, the *ZT* value of the (Sn_0.96_Cd_0.04_Te_0.99_I_0.01_)_0.94_(AgCuTe)_0.06_ sample was improved to 1.75 at 833 K.^[^ ^111^ ^]^

Due to the inherently abundant V_Sn_, developing n‐type SnTe thermoelectric materials presents a significant challenge. Hong et al. enhanced the n‐type doping rate in SnTe through PbSe alloying and achieved n‐type transport in SnTe by halogen doping. Additionally, extra Pb atoms were introduced to compensate for cation vacancies in the SnTe‐PbSe alloy, further improving *n* and electrical performance. Consequently, the n‐type (SnTe_0.98_I_0.02_)_0.6_(Pb_1.06_Se)_0.4_ sample achieved a peak *ZT* of 0.75 at 573 K.^[^ ^120^ ^]^ Similarly, Pang et al. filled V_Sn_ in SnTe through Pb alloying and sharpened the CB to enhance *µ*. By doping with iodine (I) to introduce electrons, electrical transport shifted from p‐type to n‐type. As a result, the n‐type Sn_0.6_Pb_0.4_Te_0.98_I_0.02_ sample achieved a high *ZT* of ≈0.8 at 573 K and a *ZT*_avg_ of 0.51 in the temperature range of 300–823 K.^[^ ^130^ ^]^

### 3.5 Energy Filtering

One effective approach to enhancing the thermoelectric performance of SnTe materials is the compositing method.^[^ ^156^, ^160^, ^162^, ^164^, ^169^, ^170^, ^174^, ^175^, ^178^ ^]^ By introducing interfacial energy filtering effects through composites, it is possible to not only regulate carrier behavior, particularly by altering *n* at grain boundaries to optimize carrier scattering mechanisms, but also effectively suppress *κ*, especially by enhancing phonon scattering at interfaces. Ma et al. incorporated Mg_3.2_Sb_0.6_Bi_1.4_ nanoparticles into Sn_1.03_Te, leveraging the energy filtering effect caused by the band structure mismatch with SnTe to effectively neutralize the excess holes in SnTe, significantly improving the *S*. Subsequently, multi‐walled carbon nanotubes (MWCNTs) were introduced to construct conductive pathways, optimizing *σ*. Additionally, atomic arrangement distortions at the hybrid material interfaces enhanced mid‐ to low‐frequency phonon scattering, while Bi‐doping further strengthened high‐frequency phonon scattering. Due to these multiple synergistic effects, the SnBi_0.03_Te‐1% Mg_3.2_Sb_0.6_Bi_1.4_‐0.2% MWCNT sample achieved a *ZT* value of 1.56 at 873 K.^[^ ^163^ ^]^

**Figure 15:**
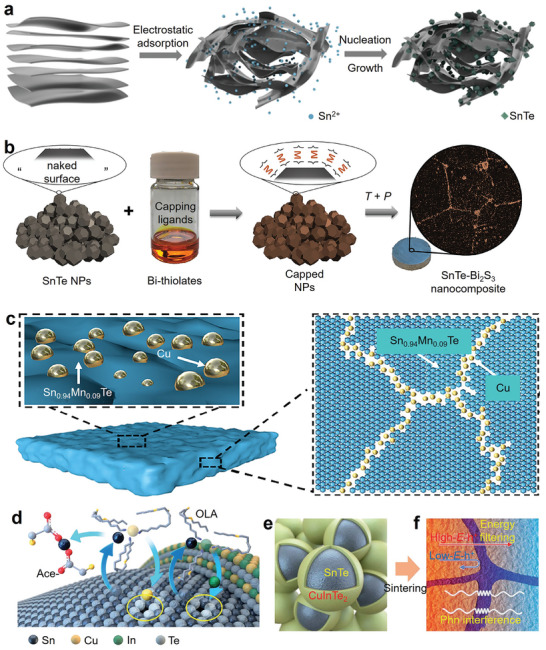
Synthesis and characterization of SnTe Nanocomposites. a) Schematic representation of the MXene/SnTe nanocomposite synthesis route. Reproduced with permission.^[^ ^168^ ^]^ Copyright 2021, Elsevier. b) Process for creating SnTe‐Bi_2_S_3_ nanocomposites. Reproduced with permission.^[^ ^171^ ^]^ Copyright 2022, Wiley. c) Schematic diagram showing Cu‐rich grain boundary features resulting from chemical plating Cu. Reproduced with permission.^[^ ^176^ ^]^ Copyright 2023, Elsevier. d) Diagram of the cation exchange reaction on SnTe powder surface. e) Schematic of the CuInTe_2_/SnTe core‐shell structure post‐exchange. f) Schematic of electrons and phonons transport characteristics in the coated grain nanocomposite. Reproduced with permission.^[^ ^165^ ^]^ Copyright 2021, Royal Society of Chemistry.

Jiang et al. employed a straightforward solvothermal method to in situ synthesize MXene/SnTe nanocomposites.^[^ ^168^ ^]^ During the synthesis process, negatively charged MXene surfaces adsorbed Sn^2+^ ions by Coulomb interactions, facilitating the growth of SnTe on MXene, as illustrated in **Figure** **15**a. The study demonstrated that the inclusion of MXene reduced *n*, optimizing the *S* and *S* ^2^ *σ*. Furthermore, the suppressed *σ* led to a reduction in *κ*_e_, consequently decreasing the *κ*. In SnTe samples with 0.6 wt.% Ti_3_C_2_T*_x_* MXene added, a *ZT*_max_ of 0.63 was achieved. Similarly, Hong et al. introduced Bi_2_O_3_ into the SnTe matrix and observed the formation of abundant nanoscale precipitates. These precipitates lowered *n* through an energy filtering effect, improved *S*, and introduced strong phonon scattering, significantly reducing *κ*_l_. As a result, SnTe‐2% Bi_2_O_3_ achieved a *ZT* value of 0.9 at 823 K.^[^ ^135^ ^]^ Li et al. fabricated Sn_0.99_In_0.01_Te‐tourmaline composites, where the introduction of tourmaline not only suppressed V_Sn_ concentrations to optimize electrical properties but also formed a secondary phase in the alloy to scatter phonons, significantly reducing *κ*_l_. Combined with the resonance effect introduced by In‐doping, the *S* ^2^ *σ* reached 25 µW cm^−1^ K^−2^ at 873 K, which is 2.5 times higher than that of pristine SnTe, while *κ*_l_ was reduced to 0.9 W cm^−1^ K^−1^.^[^ ^179^ ^]^ Lei et al. synthesized Sn_1−_ *_x_* Y*_x_* Te‐5% Cu_2_Te nanocomposites, where Cu_2_Te alloying successfully introduced interstitial Cu defects, resulting in ultralow *κ*_l_. Y‐doping induced band convergence and introduced abundant Y/Y_2_Te_3_ multiscale composite nanostructures in the matrix, significantly enhancing phonon scattering. As a result, Sn_0.97_Y_0.03_Te‐5% Cu_2_Te achieved a peak *ZT* value of 1.27 at 823 K.^[^ ^177^ ^]^

Surface modification of thermoelectric materials offers a novel strategy to simultaneously optimize charge and phonon transport.^[^ ^167^ ^]^ Chang et al. prepared ligand‐free SnTe nanoparticles under ambient pressure and then functionalized their surfaces using bismuth thiolate salts. These modified particles were thermally treated to synthesize dense SnTe‐Bi_2_S_3_ nanocomposites (as shown in Figure 15b). During the thermal treatment, the decomposition of bismuth thiolate salts doped Bi into SnTe, optimizing the *n*. Additionally, the introduced energy filtering effect enhanced the *S* and suppressed bipolar effects. Nanograins and grain boundaries reduced *κ*_l_, resulting in a peak *ZT* of 1.3 at 873 K for the SnTe‐Bi_2_S_3_ nanocomposites.^[^ ^171^ ^]^

Compared to embedded nanoparticles, coated grain boundaries can more effectively induce energy filtering. Wang et al. chemically plated Cu onto the surface of Sn_0.94_Mn_0.09_Te, forming Cu‐rich grain boundary complexes to enhance the grain boundary properties and decouple the *S* and *σ*. Simultaneously, phonon scattering at high temperatures was significantly enhanced (as shown in Figure 15c). As a result, Cu‐doped Sn_0.94_Mn_0.09_Te achieved a *ZT* value of 1.05 at 876 K.^[^ ^176^ ^]^

Constructing core‐shell structures is another effective approach for designing interfaces and introducing energy filtering effects.^[^ ^113^, ^152^, ^173^, ^241^ ^]^ Li et al. incorporated β‐Zn_4_Sb_3_ into a SnTe matrix, creating a core‐shell structure with Sb cores and ZnTe shells. This microstructure effectively compensated for V_Sn_ and achieved an ultralow *κ*_l_ of 0.48 W m^−1^ K^−1^. As a result, the SnTe‐1.5% β‐Zn_4_Sb_3_ sample attained a high *ZT* value of 1.32 at 873 K.^[^ ^153^ ^]^ Hwang et al. performed cation exchange on the surface of SnTe powders, enabling Cu and In to react with SnTe to form a CuInTe_2_ ternary coating layer (Figure 15d). After hot‐press sintering, CuInTe_2_/SnTe developed coated grain nanostructures (Figure 15e,f). This structure significantly improved the *S* ^2^ *σ* and reduced *κ*_l_ through the synergistic effects of coherent phonon scattering and energy filtering. Consequently, CuInTe_2_/SnTe nanocomposites achieved a peak *ZT* of up to 1.68 at 823 K.^[^ ^165^ ^]^

**Figure 16:**
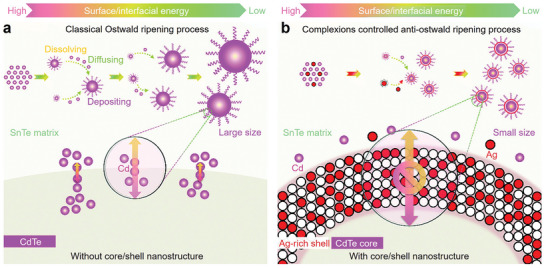
Analyzing microstructure and composition to understand inhibited Ostwald ripening. Schematic representation depicting a) the standard mechanism of Ostwald ripening and b) the impact of interfacial complexions, resulting from Gibbs adsorption of Ag at interfaces, on slowing down Ostwald ripening. Reproduced with permission.^[^ ^166^ ^]^ Copyright 2021, Royal Society of Chemistry.

Interface control is a critical scientific challenge in the design of nanocomposite thermoelectric materials. However, in multicomponent systems, supersaturated solid solutions often exhibit Ostwald ripening,^[^ ^166^ ^]^ which can degrade the thermoelectric performance of nanocomposites. Ostwald ripening is the phenomenon in which smaller particles, due to their higher surface energy, tend to dissolve, and the dissolved material is adsorbed by larger particles, leading to the growth of larger particles and the disappearance of smaller ones. The Ostwald ripening of nanoprecipitates can significantly affect the performance of thermoelectric materials.^[^ ^242^ ^]^ To address this issue, An et al. proposed a strategy to suppress Ostwald ripening through Gibbs adsorption and interfacial complexation. They designed a highly stable SnAg_0.05_Te‐*x*% CdSe thermoelectric system.^[^ ^166^ ^]^ Research revealed that the co‐alloying of Ag and CdSe facilitates the formation of high‐intensity CdTe/Ag core‐shell nanoprecipitates, with the Ag‐rich interfacial composite structure playing a critical role in suppressing precipitate growth, as shown in **Figure** **16**. During the cooling process of the prepared samples, the initial stage of the supersaturated solid solution generates metastable precipitates of varying sizes, placing the system in a thermodynamically nonequilibrium state and increasing the total surface free energy. In Ag‐free systems, to reduce free energy, CdTe precipitates dissolve or grow, decreasing the total interfacial area. The dissolved precipitates form a supersaturated solution and aggregate on larger particles, ultimately reducing the number of precipitates and increasing their average size (Figure 16a). However, in Ag‐containing samples, the Ag‐rich interfacial composite structure effectively slows the Ostwald ripening process by reducing interfacial energy, suppressing precipitate growth. After high‐temperature annealing, the number density of CdTe precipitates in Ag‐containing samples remained stable (Figure 16b). This unique defect structure enables full‐spectrum phonon scattering, reducing the *κ*_l_ to as low as 0.36 W m^−1^ K^−1^ at 873 K. Furthermore, the dual combination of Ag and CdSe results in band flattening and the full convergence of the L and Σ bands, thereby enhancing weighted mobility. Ultimately, SnAg_0.05_Te‐6% CdSe achieved a peak *ZT* of 1.5 at 873 K.

## 4 2D SnTe

In certain cases, 2D materials exhibit superior thermoelectric performance compared to their bulk counterparts due to various quantum phenomena arising from symmetry breaking.^[^ ^243^, ^244^, ^245^, ^246^, ^247^ ^]^ For example, based on DFT, the peak *ZT* of a SnSe monolayer at 700 K (3.27) is seven times higher than its bulk counterpart.^[^ ^248^ ^]^ At 500 K, the theoretical *ZT* values for monolayers of GeTe, SnTe, and PbTe are calculated to be 1.26, 2.61, and 5.91, respectively.^[^ ^249^ ^]^ Theoretical *ZT* values for “buckled” β‐SnX (X = S, Se, Te) monolayers are as high as 2.45 and 3.81 at 600 and 900 K, respectively.^[^ ^250^ ^]^ For n‐type monolayer SnTe with a buckled structure, *ZT* is predicted to exceed 1.6 at 900 K.^[^ ^251^ ^]^ Theoretical predictions suggest that Mn‐doped 2D SnTe can achieve a *ZT* of 2.24 at 900 K.^[^ ^185^ ^]^ Experimental results indicate that due to the high *S* and low *κ* of nanostructured 2D SnTe, its *ZT* reaches 0.17 at room temperature, significantly higher than the bulk *ZT* (0.005).^[^ ^182^ ^]^ Therefore, systematically studying and exploring the transport behavior and evolution of thermoelectric performance in ultrathin 2D Sn‐based materials is highly meaningful.

### 4.1 Monolayer

In monolayer SnTe systems, common structures include buckled and wrinkled configurations. Lubis et al. investigated the thermoelectric properties of monolayer and bilayer SnTe with buckled structures using DFT simulations and the Boltzmann transport equation (BTE).^[^ ^251^ ^]^ Research revealed that monolayer SnTe exhibits a hexagonal honeycomb structure with periodic buckled topology, classifying it as an indirect bandgap semiconductor with a bandgap of 1.87 eV. The CBM demonstrates band convergence, resulting in exceptionally high *S*. Similar to monolayer SnTe, the band structure of bilayer SnTe shows analogous features. However, the additional bands from the second layer, concentrated near the Fermi level, reduce the bandgap to 1.31 eV, leading to lower *S* compared to the monolayer. Calculations further indicate that both *σ* and *κ*_e_ are lower in bilayer SnTe than in the monolayer. Overall, the ideal *ZT* values for both monolayer and bilayer SnTe are significantly higher than those of their bulk counterparts, with monolayer SnTe exhibiting superior *ZT* values compared to the bilayer. This suggests that reducing SnTe to monolayer thickness can markedly enhance its thermoelectric performance. The *ZT* value of n‐type buckled monolayer SnTe is expected to exceed 1.6 at 900 K. Liu et al. investigated the thermoelectric properties of monolayer SnTe using first‐principles calculations and the BTE.^[^ ^249^ ^]^ The results indicate that monolayer SnTe is an indirect bandgap semiconductor with a relatively small *m* ^*^, leading to higher *µ* and *σ*. Additionally, due to its low *v* and high *γ*, SnTe exhibits a low *κ*_l_. As a result, its *ZT* value can reach up to 2.61 at 500 K.

**Figure 17:**
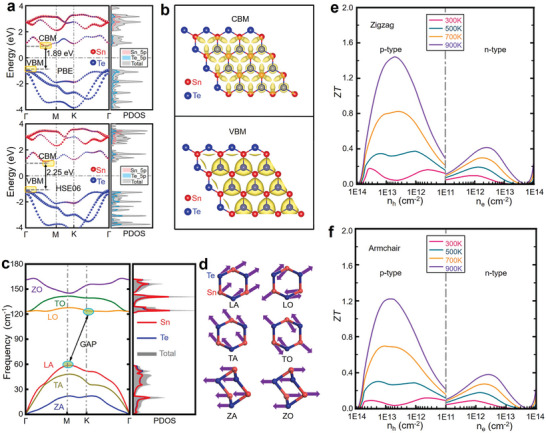
Characterization of SnTe monolayers. a) Electronic band structures and partial density of states (PDOS) of SnTe monolayer calculated using Perdew‐Burke‐Ernzerhof (PBE) and hybrid density functional (HSE06). b) Spatial charge densities for the valence band maximum (VBM) and conduction band minimum (CBM) of the SnTe monolayer. c) Phonon dispersion relations and phonon DOS (PhDOS) for the SnTe monolayer. d) Vibrational modes of acoustic (LA, TA, ZA) and optical (LO, TO, ZO) phonon branches at the Γ point. e,f) *ZT* values of the SnTe monolayer along the zigzag‐ and armchair‐ directions as a function of carrier concentration at various temperatures. Here *n*_h_ and *n*_e_ represent the hole carrier concentration in p‐type materials and electron carrier concentration in n‐type materials. Reproduced with permission.^[^ ^252^ ^]^ Copyright 2023, Elsevier.

Tang et al. systematically investigated the thermoelectric properties of monolayer SnTe with a corrugated structure using first‐principles calculations and BTE.^[^ ^252^ ^]^ The electronic band structure and PDOS of monolayer SnTe were calculated using both Perdew‐Burke‐Ernzerhof (PBE) and hybrid density functional (HSE06) methods, with results shown in **Figure** **17**a. Both methods revealed a similar trend in the electronic band structure: the VBM is located at the Γ point and is relatively flat, indicating a high valley degeneracy, while the CBM lies along the Γ‐M direction, confirming that monolayer SnTe is an indirect bandgap semiconductor. The calculated bandgaps were 1.89 and 2.25 eV using PBE and HSE06 functionals, respectively. The PDOS in Figure 17a exhibits a sharp peak at the VBM, suggesting that monolayer SnTe holds significant potential for achieving high thermoelectric performance through p‐type doping. Additionally, the charge density distribution shown in Figure 17b indicates that the CBM mainly originates from the hybridization of Sn‐5p and Te‐5p orbitals, while the VBM is predominantly contributed by Te‐5p orbitals.

Figure 17c presents the phonon dispersion curve and phonon density of states (PhDOS) for monolayer SnTe.^[^ ^252^ ^]^ The three acoustic phonon modes comprise in‐plane vibrational modes (longitudinal acoustic, LA, and transverse acoustic, TA) and an out‐of‐plane vibrational mode (flexural, ZA). The in‐plane vibrational modes exhibit linear dispersion, while the out‐of‐plane mode shows quadratic dispersion. The flat acoustic phonon dispersion curves in the low‐frequency range (0–60 cm^−1^) indicate low phonon group velocities, which are favorable for achieving low *κ*_l_. The three optical branches of SnTe monolayer, namely in‐plane modes (TO and LO) and out‐of‐plane mode (ZO), exhibit relatively high frequencies in the range of 120–160 cm^−1^. PhDOS analysis reveals that both Sn and Te atoms contribute comparably to the acoustic and optical branches. The sharp PhDOS peaks in the low‐frequency region further support the reduction of *κ*_l_. Figure 17d illustrates two vibrational modes (TA/LA/TO/LO and ZA/ZO) and the vibrational patterns of each atom at the Γ point. The figure demonstrates that Sn and Te atoms vibrate parallel or antiparallel and perpendicular to the monolayer SnTe plane, respectively. The strong phonon‐phonon scattering observed in these modes effectively suppresses phonon transport, contributing to the realization of low *κ*_l_.

Figure 17e,f depicts the relationship between the *ZT* values of monolayer SnTe along the zigzag and armchair directions and *n*, respectively.^[^ ^252^ ^]^ For both n‐type and p‐type SnTe monolayers, the *ZT* values initially increase with *n* and then decrease. Notably, the *ZT* values for p‐type SnTe are higher than those for n‐type SnTe, likely due to the higher *µ*. At 300 K, the *ZT*_max_ values for p‐type SnTe monolayers are 0.18 and 0.16 along the zigzag and armchair directions, respectively. At 900 K, the *ZT*_max_ values increase to 1.44 and 1.22, respectively. These results suggest that monolayer SnTe is a promising candidate for p‐type thermoelectric materials.

Due to quantum confinement effects, 2D tin chalcogenides exhibit unique structural and electronic properties, resulting in lower *κ*_l_ and enhanced *S* from the increased density of electronic states near the Fermi level. As a result, they demonstrate superior thermoelectric performance compared to their bulk counterparts. Xiong et al. useed DFT and Boltzmann transport theory to calculated that biaxial strain can effectively alter the DOS near the Fermi level in 2D SnTe, thereby improving both *σ* and *S*.^[^ ^253^ ^]^ Additionally, their study revealed that V_Sn_ form easily in 2D SnTe, leading to p‐type conductivity. Iodine (I) and arsenic (As) were identified as effective dopants for n‐type and p‐type 2D SnTe, respectively.

Biswas et al. investigated the thermoelectric properties of 3D transition metal (3D‐TM) doped 2D SnTe through first‐principles calculations combined with Boltzmann transport theory.^[^ ^185^ ^]^ Their study revealed that the hexagonal β′‐SnTe phase possesses a layered structure, with the unit cell consists of two sublayers. Each sublayer contains one Sn atom and one Te atom, firmly bonded by Sn─Te covalent bonds. The two centrosymmetric Sn‐Te sublayers are connected by weaker Sn─Sn van der Waals interactions, ensuring good lattice dynamical stability. V‐ and Mn‐doping reduced both the formation energy and binding energy, confirming the stability of the doped systems. Replacing 25% of Sn with V or Mn led to a reduction in elastic constants and an increase in *m* ^*^, resulting in moderate intrinsic *µ* and a slight enhancement in the *S*. V and Mn doping also strengthened phonon‐phonon scattering, significantly reducing the *κ*_l_. Ultimately, Mn‐doped 2D SnTe achieved a remarkable *ZT* value of 2.24 at 900 K, demonstrating excellent thermoelectric performance.

In experiments, Singh et al. synthesized bulk and 2D SnTe using flame melting and liquid‐phase exfoliation methods, respectively.^[^ ^182^ ^]^ Their study revealed that 2D SnTe exhibits a larger bandgap and introduces more interfaces. These interfaces enhance phonon scattering rather than electron scattering by filtering out low‐energy carriers, resulting in higher *S* for 2D SnTe compared to its bulk counterpart. Notably, monolayer and bilayer SnTe demonstrated the highest *S* values. The size reduction introduced a higher intensity of interfaces and boundaries, which served as effective phonon scattering centers. Moreover, quantum confinement effects shortened the phonon mean free path, leading to significantly lower *κ* in 2D SnTe compared to the bulk material. As a result, the room‐temperature *ZT* value of 2D SnTe reached 0.17, 34 times higher than that of the bulk material (0.005). Ju et al. fabricated porous SnTe nanosheets using an anion exchange reaction. The introduced nanointerfaces, porosity, and defects significantly reduced *κ*_l_ while enhancing *S* compared to bulk SnTe. This approach achieved a *ZT* value of 1.1 at 923 K without the need for atomic doping processes.^[^ ^181^ ^]^

### 4.2 Van Der Waals Gap

It has been reported that anisotropic van der Waals (vdWs) gaps play a critical role in enhancing the *S* ^2^ *σ* and reducing the *κ*_l_ of SnTe‐based thermoelectric materials.^[^ ^125^ ^]^ At room temperature, SnTe exists in the β‐SnTe phase, while under high pressure or in ultrathin samples, the γ‐SnTe phase can emerge.^[^ ^254^ ^]^ Consequently, introducing local atomic‐scale phase transitions has become an effective strategy for optimizing the thermoelectric performance of β‐SnTe. Zhang et al. reported the formation of high‐density γ‐SnTe ultrathin nanosheets within the β‐SnTe matrix. These nanosheets are connected to the matrix interface through weak interlayer vdWs interactions, leading to increased atomic spacing and introducing local lattice distortions that significantly enhance phonon scattering. The VB offset between γ‐SnTe and the β‐SnTe matrix is only ≈0.05 eV, allowing effective carrier transport across the interface. The synergistic effects of interfacial phonon scattering and band alignment show great promise for improving the thermoelectric performance of β‐SnTe.^[^ ^255^ ^]^

**Figure 18:**
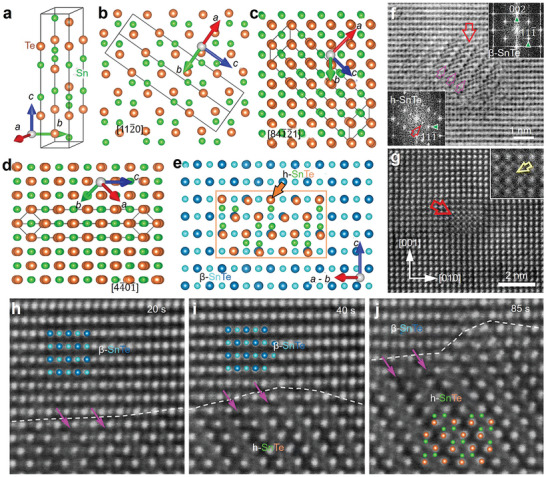
Structural properties of precipitate in SnTe matrix. a) A unit cell of rhombohedral h‐SnTe and its projections along the crystallographic directions of b) [$11\bar{2}0$], c) [$84\bar{12}1$], and d) [$4\bar{4}0\bar{1}$]. Reproduced with permission.^[^ ^256^ ^]^ Copyright 2024, Royal Society of Chemistry. e) Structure model showing the overlap of the h‐SnTe with the β‐SnTe matrix along the [$1\bar{1}0$] β‐SnTe zone axis. f) ABF‐STEM images of the β‐SnTe sample along the [$1\bar{1}0$] β‐SnTe zone axis, with the region of interest (ROI) indicated by red arrows and the vdWs gaps shown by purple arrows. The FFT patterns of β‐SnTe and h‐SnTe are inserted, highlighting extra spots in h‐SnTe with a red arrow. g) HAADF‐STEM image of the β‐SnTe sample containing the ROI under electron beam irradiation (100s). h–j) Atomic‐resolution HAADF‐STEM images illustrating the formation of the h‐SnTe precipitate in the β‐SnTe matrix along the [$1\bar{1}0$] zone axis, captured at 20, 40, and 85 s under continuous electron‐beam irradiation, with structural models of β‐ and h‐SnTe superposed and vdWs gaps formation in h‐SnTe marked by oblique arrows. Reproduced with permission.^[^ ^256^ ^]^ Copyright 2024, Royal Society of Chemistry.

Zhang et al. utilized electron beam irradiation in aberration‐corrected STEM to induce the formation of nanoscale h‐SnTe structures with interlayer vdWs gaps within the β‐SnTe matrix.^[^ ^256^ ^]^ The h‐SnTe unit cell exhibits a hexagonal configuration with triangular symmetry, composed of six Sn atoms and six Te atoms, as shown in **Figure** **18**a,b depicts the projection of the h‐SnTe structure along the [$11\bar{2}0$] zone axis. Figure 18c,d shows projections of the h‐SnTe structure along the [$84\bar{12}1$] and [$4\bar{4}0\bar{1}$] zone axes. For the h‐SnTe structure, the calculated rotation angle between the [$11\bar{2}0$] and [$84\bar{12}1$] directions is 45°, while the angle between the [$11\bar{2}0$] and [$4\bar{4}0\bar{1}$] directions is 90°. These align with the angles between the ⟨100⟩ zone axis and the ⟨110⟩ zone axis of β‐SnTe, confirming that the same h‐SnTe structure is formed under electron beam irradiation along the ⟨110⟩ or ⟨100⟩ zone axes of β‐SnTe. Figure 18e illustrates the overlap of the h‐SnTe structure with the β‐SnTe matrix when observed along the [$1\bar{1}0$]_β‐SnTe_ zone axis.

Figure 18f displays an annular bright field (ABF) STEM image of the region of interest (ROI, marked by a red arrow) after 100 s of electron beam irradiation, revealing periodically arranged vdWs gaps (indicated by purple arrows) distributed along the ⟨112⟩ direction of β‐SnTe. The fast Fourier transform (FFT) of the ROI (bottom‐left inset) shows additional spots (highlighted by red arrows) compared to the FFT of pristine β‐SnTe (top‐right inset), indicating lattice modulation induced by electron beam irradiation in SnTe. Figure 18g presents a high‐angle annular dark field (HAADF) STEM image of the ROI (red arrow) recorded after 100 s of electron beam irradiation. Additional atomic columns are clearly observed within the ROI. The inset demonstrates that high‐intensity Te columns are mostly retained within the normal β‐SnTe lattice, while low‐intensity Sn columns (marked by yellow arrows) are surrounded by four adjacent Te columns. Figure 18h–j illustrate the formation process of h‐SnTe precipitates along the [$1\bar{1}0$] zone axis within the β‐SnTe matrix. Purple diagonal arrows denote the formation of vdWs gaps in h‐SnTe. The electron beam irradiation predominantly displaces Sn atoms within the SnTe lattice, leading to the formation of h‐SnTe structures. This discovery provides a novel strategy for designing nanoscale heterostructures in chalcogenides to optimize the thermoelectric performance of materials.

### 4.3 Thin Film

Thermoelectric SnTe thin films have garnered significant attention for their potential applications in strategic emerging industries such as new energy and electronic information.^[^ ^257^ ^]^ With the growing demand for high‐performance electronic devices, investigating the growth mechanisms of SnTe thin films and enhancing their thermoelectric properties have become focal points of research.^[^ ^188^, ^192^ ^]^ Hua et al. prepared SnTe (111) thin films using molecular beam epitaxy (MBE) and studied defect engineering. The results revealed that V_Sn_ and Sn interstitials (Sn_i_) are the primary defects governing electronic transport. Moreover, increasing the substrate temperature reduced the density of V_Sn_ in the thin films, thereby improving *µ*.^[^ ^189^ ^]^

Ashfaq et al. studied the effect of annealing temperature on the *S* ^2^ *σ* of Sn_0.7_Sr_0.3_Te thin films and found that Sr‐doping and post‐annealing treatment could modify the band structure and introduce energy filtering effects at grain boundaries, significantly enhancing thermoelectric performance. The Sn_0.7_Sr_0.3_Te thin films achieved a maximum *S* ^2^ *σ* of 29.4 µW m^−1^ K^−2^ at 523 K.^[^ ^190^ ^]^ Shokralla et al. prepared Sr‐doped SnTe thin films where carrier localization at grain boundaries, particularly due to energy filtering effects, led to improved *S*. These films exhibited a significantly enhanced *S* ^2^ *σ* of 38.9 µW m^−1^ K^−2^ at 425 K.^[^ ^191^ ^]^ Zhu et al. investigated the thermoelectric properties of SnTe thin films with varying thicknesses (67–610 nm) and found that a thickness of 224 nm yielded the highest *ZT* of ≈0.27 at 600 K due to the combined effects of grain morphology and defect scattering. The study also showed that the *ZT* of SnTe thin films was notably higher than that of bulk materials, demonstrating that reducing dimensionality is an effective strategy for controlling *ZT*.^[^ ^180^ ^]^

## 5 Device

In the field of SnTe‐based thermoelectric research, the most significant progress over the past five years has been the preliminary exploration of the performance of SnTe‐based TEDs.^[^ ^160^, ^183^, ^258^ ^]^ This breakthrough has opened new prospects for the practical application of SnTe‐based thermoelectric technology. Studies have not only revealed critical issues during the assembly process of SnTe‐based TEDs, such as increased interfacial resistance and material stability, but also provided valuable insights and guidance for further optimization of SnTe‐based materials.^[^ ^39^, ^118^ ^]^ These findings offer an important reference for future researchers in optimizing materials and device designs, laying a solid foundation for improving SnTe‐based TED performance and expanding its application scope. This work holds significant academic and engineering value.

### 5.1 Device Fabrication and Testing

The assembly of TEDs plays a critical role in determining their performance, particularly in ensuring performance and long‐term stability.^[^ ^259^ ^]^ Traditional rigid TEDs typically comprise ceramic substrates, thermoelectric legs, electrode sheets connecting the thermoelectric legs, wires, and various soldering materials. The ceramic substrate provides structural support and facilitates thermal management, while the thermoelectric legs perform the core function of converting thermal energy into electrical energy through the thermoelectric effect. Electrode sheets enable electrical connections between the thermoelectric legs and external circuits, and wires are used for current transmission. During the assembly process, soldering materials play an essential role in ensuring good contact and electrical conductivity among the components.

**Figure 19:**
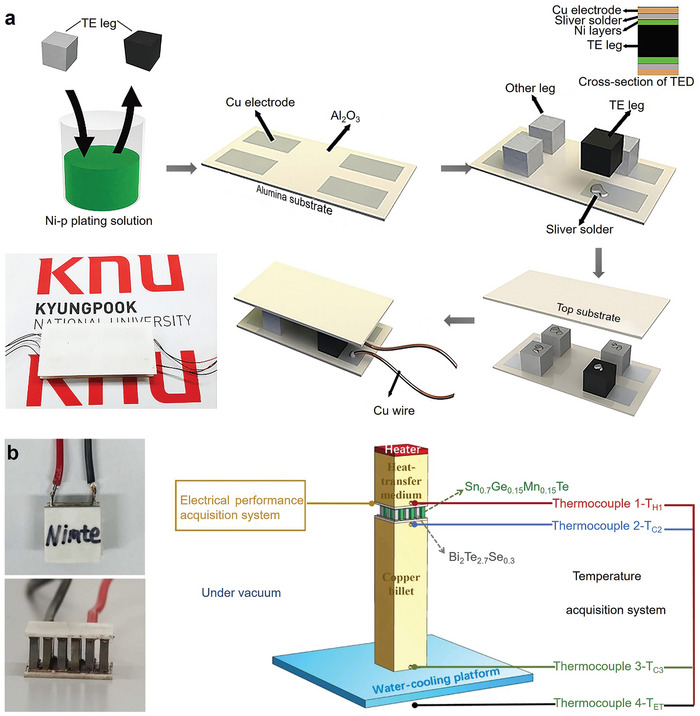
Fabrication and testing of a SnTe‐based thermoelectric device (TED). a) Schematic representation of the fabrication process for the single‐leg thermoelectric device. Reproduced with permission.^[^ ^97^ ^]^ Copyright 2024, American Chemical Society. b) Optimal images of the fabricated (Sn_0.96_Sb_0.04_Te)_0.7_(Ge_0.5_Mn_0.5_Te)_0.3_/Bi_2_Te_2.7_Se_0.3_ + 0.01 wt% BiCl_3_ thermoelectric module, along with a schematic diagram of the custom thermoelectric testing system. Reproduced with permission.^[^ ^107^ ^]^ Copyright 2022, Elsevier.

Kihoi et al. systematically studied the thermoelectric performance of single‐leg TEDs using pristine SnTe samples and multi‐cation alloyed SnTe samples, where the ternary CuInTe_2_ phase was introduced into the SnTe matrix through equimolar multi‐cation alloying. **Figure** **19**a illustrates the TED fabrication process.^[^ ^97^ ^]^ The TED was constructed using Al_2_O_3_ ceramic plates as the substrate and Cu electrodes as the electrode sheets. Ni‐coated thermoelectric legs were soldered to the Cu electrodes using Ag‐based solder, and commercial Ag paste was used to connect Cu wires to the Cu electrodes, forming a TED with four thermoelectric legs. Results showed that the TED assembled with multi‐cation alloyed SnTe produced a maximum output power (*P*) of ≈337.6 µW, which was ten times higher than that of the pristine SnTe‐based TED.

Zhang et al. utilized SnTe‐GeMnTe_2_ medium‐entropy alloy as the p‐type legs and Bi_2_Te_2.7_Se_0.3_ as the n‐type legs. All thermoelectric legs were polished to a cross sectional area of 1.4 × 1.4 mm^2^ and a length of 5.0 mm, achieving a fill factor of 39%. High‐temperature Sn_5_Pb_92.5_Ag_2.5_ solder was used to bond the legs to copper electrodes, assembling a TED with 17 pairs of thermoelectric legs. The *η* and output power density (*ω*) were measured using a custom‐built testing system, as illustrated in Figure 19b. The TED achieved a *η* of 6.1% and a *ω* of 0.43 W cm^−2^ at a Δ*T* of 350 K.^[^ ^107^ ^]^

### 5.2 Performance Evaluation and Optimization

**Figure 20:**
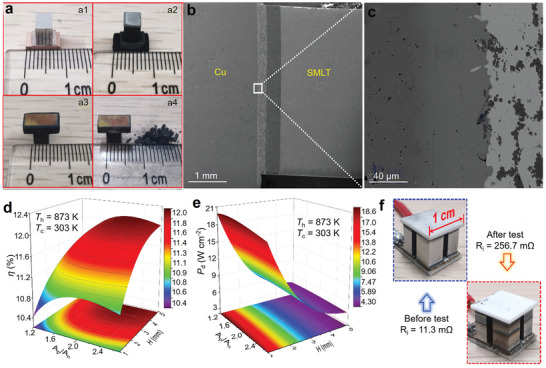
Characterization and performance of the SnTe‐based thermoelectric module. a) Connection of the Cu electrode to the p‐type Sn_0.88_Mn_0.12_Li_0.01_Te leg using AgCu‐based solder. b) Scanning electron microscopy (SEM) image of the Cu electrode and p‐type leg joint by transient liquid phase (TLP) bonding. c) Back‐scattered electron (BSE) images of the TLP bonding joint after treatment at 873 K for 24 h. d) Simulated maximum *η* and e) output power density (*P*_d_) as functions of the leg area ratio (*A*_p_/*A*_n_) and leg height (*H*) for the module. f) Optical images showing the module before and after testing. Reproduced with permission.^[^ ^84^ ^]^ Copyright 2022, Elsevier.

The assembly of a TED goes beyond the mere physical connection of its components.^[^ ^260^ ^]^ It involves optimizing material compatibility, interface resistance, thermal resistance, and the distribution of mechanical stress, all of which significantly impact the overall thermoelectric performance and stability of the device.^[^ ^96^, ^160^ ^]^ During the assembly of TEDs, barrier layers are typically constructed on the surface of thermoelectric legs to prevent reactions between the solder and thermoelectric materials. For instance, when using Ag_56_Cu_22_Zn_17_Sn_5_ solder to connect p‐type thermoelectric legs with Cu electrodes (**Figure** **20**a), severe volatilization of the thermoelectric material, powdering of the solder, and loose structures surrounding the Cu electrode are observed post‐welding. To address this issue, Guo et al. employed a one‐step sintering method to fabricate 304 stainless steel/Sn composite barrier layers and innovatively utilized transient liquid phase (TLP) bonding technology, enabling low‐temperature joining and high‐temperature operation at the hot side.^[^ ^84^ ^]^ By inserting Sn foil between the Cu electrode and the p‐type thermoelectric leg (Figure 20b), a continuous, pore‐free high‐quality joint was achieved. After annealing at 873 K for 24 h, Sn was uniformly distributed within the Cu and Ag phases (Figure 20c), forming a solid solution joint capable of withstanding operating temperatures up to 873 K, with a room‐temperature shear strength of 77.3 MPa. Numerical simulations were conducted to optimize the geometry of thermoelectric legs and study the thermoelectric transport properties of the TED (Figure 20d,e). The results indicated that for a total cross sectional area of the thermoelectric legs *A*_pn_ = *A*_p_ + *A*_n_ = 26 mm, the maximum output power density (*P*_d_) occurred at *A*_p_/*A*_n_ = 1.5, while the highest *η* was observed at *A*_p_/*A*_n_ = 1.9. As the height (*H*) of the thermoelectric legs increased, the *η* improved, but the internal resistance increased, leading to a decrease in performance. A TED was assembled using p‐type Sn_0.88_Mn_0.12_Li_0.01_Te (4 × 4 × 5.5 mm^3^) and n‐type Yb_0.25_Co_4_Sb_12_ (3.2 × 3.2 × 5.5 mm^3^) thermoelectric materials, with an approximate height of 4.5 mm (Figure 20f). Performance testing revealed that the room‐temperature internal resistance increased from 11.3 to 256.7 mΩ, which was significantly higher than the theoretical value of 6.9 mΩ, primarily due to microcracks in the Sn_0.88_Mn_0.12_Li_0.01_ thermoelectric material at the hot‐side joint caused by rapid cooling. Ultimately, the fabricated TED demonstrated an *η* of ≈5.4% and a high *ω* of 1.9 W cm^−2^ under a Δ*T* of 530 K.

**Figure 21:**
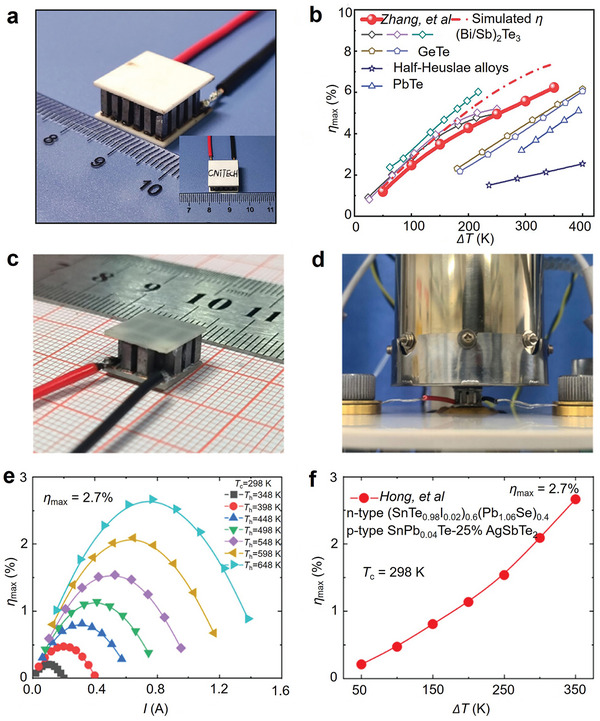
Characterization and performance of the SnTe‐based thermoelectric module. a) Optical images of the 17‐couple SnTe‐based thermoelectric module. b) Measured *η* as a function of Δ*T*, along with comparative data from other thermoelectric modules. Reproduced with permission.^[^ ^40^ ^]^ Copyright 2022, Wiley. c) Photograph of the assembled all‐SnTe‐based seven‐pair TED. d) Mini‐PEM testing for power generation performance. e) Measured *η* as a function of current (*I*) at various hot‐side temperatures (*T*_h_), with the cold‐side temperature (*T*_c_) held at ≈298 K. f) Maximum *η* at different Δ*T*s. Reproduced with permission.^[^ ^120^ ^]^ Copyright 2024, American Chemical Society.

Zhang et al. successfully fabricated a TED with 17 pairs of p‐n thermoelectric legs using Cd_0.02_(Sn_0.59_Pb_0.15_Ge_0.2_Sb₀_0.06_)_0.98_Te as the p‐type legs and Bi_2_Te_2.7_Se_0.3_ + 0.097 wt% BiCl_3_ as the n‐type legs (**Figure** **21**a), and evaluated its thermoelectric performance (Figure 21b).^[^ ^40^ ^]^ The results showed that the *η* increased with the hot‐side temperature, reaching a peak of 6.3% at Δ*T* = 350 K. Compared to conventional Bi_2_Te_3_‐based TEDs, the *η* was improved in the range of 250–350 K. The *η* of 6.3% is also competitive with TEDs based on GeTe, PbTe, and half‐Heusler materials. Multi‐physics simulations predicted a maximum *η* of 7.4%, ≈18% higher than the experimentally measured value. This indicates that SnTe‐based TEDs hold significant potential for further improvement. Future advancements could be achieved through topology optimization, reducing interface resistance, or selecting more efficient n‐type materials to enhance *η*.

Xia et al. proposed a vacancy‐based entropy engineering strategy by introducing AgSbTe_2_ into SnTe, removing Ag_2_Te to create V_Sn_, and incorporating Pb and Ge into cation sites to enhance configurational entropy, thereby leveraging the core effects of high‐entropy alloys.^[^ ^261^ ^]^ They fabricated and tested a single‐stage dual‐couple TED composed of p‐type AgSb_3_Pb_2_Ge_2_Sn_6_Te_15_ thermoelectric legs and n‐type PbTe thermoelectric legs. The TED achieved a high *η* of 9.3% under a Δ*T* of 478 K.^[^ ^262^ ^]^ Hong et al. developed an n‐type (SnTe_0.98_I_0.02_)_0.6_(Pb_1.06_Se)_0.4_ thermoelectric material and, for the first time, constructed an all‐SnTe‐based TED using this n‐type material paired with p‐type SnPb_0.04_Te‐25%AgSbTe_2_ thermoelectric legs (Figure 21c). Using the Mini‐PEM instrument, the *η* was estimated under Δ*T* conditions (Figure 21d,e). The results showed that at Δ*T* = 350 K, the maximum *η* reached 2.7% (Figure 21f).^[^ ^120^ ^]^

### 5.3 Flexible Device

**Figure 22:**
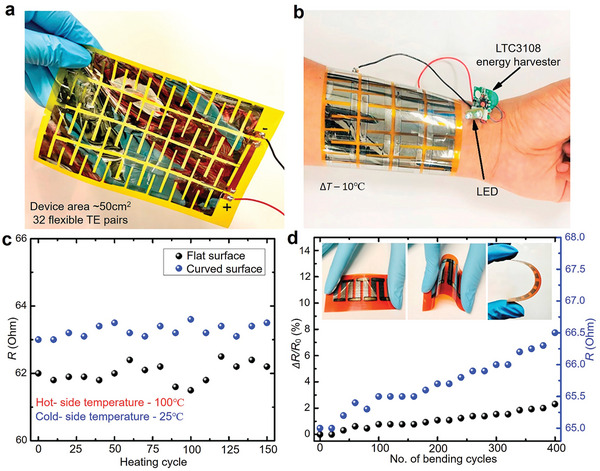
Characterization of flexible SnTe–based thin film thermoelectric device (TED). a) Image of the large‐area flexible TED featuring 32 thermoelectric pairs. b) Real‐time demonstration of the TED functioning as a wearable power source by illuminating an LED with body heat. c) Variation in internal resistance with the number of heating cycles. d) Internal resistance changes of the TED with the number of bending cycles (bending radius = 3 cm) at ambient temperature. Reproduced with permission.^[^ ^267^ ^]^ Copyright 2020, Elsevier.

Wearable electronic devices enable multifunctional applications by real‐time monitoring of human activities.^[^ ^263^, ^264^ ^]^ Self‐powered nanotechnology, which converts body heat into electricity, provides a solution for ultra‐flexible, cost‐effective, battery‐free devices, making it a focal point of current research.^[^ ^265^, ^266^ ^]^ Karthikeyan et al. developed a TED based on SnTe‐PbTe thin films, which achieved a maximum *P*_d_ of 8.4 mW cm^−2^.^[^ ^267^ ^]^ The TED was fabricated using thermal evaporation technology, in which 100 nm thick n‐type PbTe and p‐type SnTe arrays were deposited as thermoelectric legs on a clean, flexible polyimide substrate. Aluminum was used as the contact material to establish electrical connections, resulting in a large‐area TED with an effective area of 50 cm^2^ and 32 pairs of p‐n thermoelectric legs (**Figure** **22**a). This device was wrapped around a human wrist to power a low‐grade LED light (Figure 22b). The generated voltage corresponded to the Δ*T* = 10 °C between the wrist and the ambient environment, which was stabilized using an LTC 3108 energy harvesting circuit. To evaluate the long‐term performance of the TED, researchers analyzed its stability during heating and cooling cycles by continuously measuring changes in internal resistance. Figure 22c shows the device stability after 150 thermal cycles, indicating no significant changes in internal resistance on either planar or curved surfaces. Figure 22d demonstrates the flexibility of the thermoelectric film on polyimide substrate, and further studies examined the stability of the internal resistance under varying bending radii (3–5 cm). Experimental results revealed that the internal resistance variation was less than 2.5% after 400 cycles under both planar and bent conditions, suggesting that the as‐fabricated TED is well‐suited for practical applications.

### 5.4 SnTe as Interlayer Material

In TED devices, the reliability of internal interconnections is critical to the overall performance and lifespan of the system. The maximum *P* of the device is directly influenced by the total internal resistance, including both the intrinsic resistance of the thermoelectric legs and the contact resistance at the interface between the thermoelectric legs and the electrodes. To achieve low total internal resistance, it is essential to ensure a reliable low‐resistance contact between the thermoelectric legs and the electrodes.^[^ ^268^ ^]^ Therefore, robust electrode connections are fundamental to the stable operation of thermoelectric waste heat recovery systems, making the selection of appropriate electrode materials and contact designs crucial.

SnTe is a suitable p‐type compound for mid‐temperature applications and serves as an interlayer material between electrodes and thermoelectric materials. For example, Jayachandran et al. observed that direct contact between Na_0.02_Pb_0.98_Te thermoelectric legs and materials such as Ni, Fe, and Co leads to poor interface bonding, “poisoning” of the thermoelectric material (i.e., contamination of the thermoelectric material by electrode elements during high‐temperature operation, which alters its performance), and increased interface contact resistance. To address these issues, introducing a SnTe interlayer between Na_0.02_Pb_0.98_Te and the metal contacts significantly reduces contact resistance.^[^ ^269^ ^]^ Although the poisoning phenomenon of Na_0.02_Pb_0.98_Te with Ni still exists, this treatment does not negatively impact the mechanical integrity of the Fe contact interface. The Co/SnTe/Na_0.02_Pb_0.98_Te samples exhibit lower contact resistance, improved adhesion, and avoid the poisoning of Na_0.02_Pb_0.98_Te. Moreover, due to the low thermal expansion coefficient of SnTe and its good compatibility with Co, the addition of a (Co + 75 vol% SnTe) buffer layer further enhances the mechanical stability of the Co/Na_0.02_Pb_0.98_Te connection. This technique reduces the contact resistance to below 40 µΩ cm^2^, and even after annealing at 723 K for 170 h, the contact resistance remains below 50 µΩ cm^2^, preserving the thermoelectric performance of Na_0.02_Pb_0.98_Te.^[^ ^269^ ^]^

Since Bi_2_Te_3_‐based thermoelectric materials and Sn‐based solders operate within a similar temperature range,^[^ ^270^ ^]^ Sn‐based solders are commonly used to interconnect these materials with electrode substrates. However, during prolonged use, diffusion at the interface between the solder and thermoelectric material intensifies, with brittle SnTe being identified as a primary cause of electrode failure. To investigate this issue, Liu et al. subjected the hot‐side electrode to aging treatment at 150 °C, and studied the diffusion behavior and crack formation process at the Bi_2_Te_3_/Sn‐based solder interface.^[^ ^271^ ^]^ The results revealed that the diffusion of Sn and Te led to the formation of Bi_2_Te_3_ and SnTe, with diffusion‐induced cracks primarily occurring at the Bi_2_Te_3_/SnTe and SnTe/Sn interfaces. The former was associated with phase transitions induced by Sn atom diffusion, while the latter resulted from the generation and growth of Kirkendall voids.^[^ ^271^ ^]^ The brittleness of the Bi‐rich phase further exacerbated crack propagation. Electrode shear tests confirmed that cracks were the weak points in TED electrodes. To mitigate this issue, it is recommended to avoid direct soldering and to introduce a protective barrier layer at the interface.

**Figure 23:**
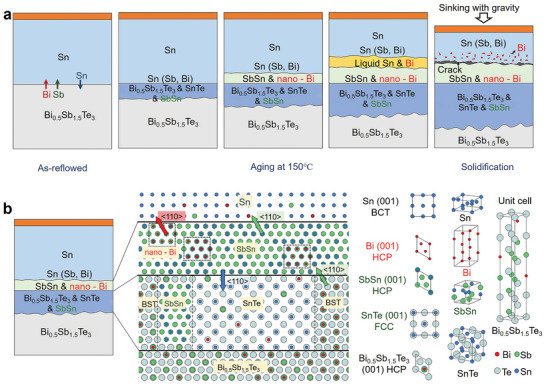
Evolution of SnTe intermetallic compound layers at the Bi‐Sb‐Te/Sn interface. a) Schematic representation of the development of intermetallic compound (IMC) layers and phase compositions at the Bi‐Sb‐Te/Sn interface during aging. b) Schematic illustration of the atomic arrangement and growth direction of phases within the IMC layers at the β‐Sn/Sn‐Sb/Sn‐Bi‐Sb‐Te/Bi‐Sb‐Te multi‐interfaces. Reproduced with permission.^[^ ^272^ ^]^ Copyright 2023, Elsevier.

Liu et al. investigated the microstructural evolution of the SnTe intermetallic compound (IMC) layer at the Bi‐Sb‐Te/Sn interface during aging and analyzed the failure mechanisms in conjunction with diffusion processes, as illustrated in **Figure** **23**a.^[^ ^272^ ^]^ The study revealed that during isothermal aging, Bi, Sb, Te, and Sn elements diffused along their respective concentration gradients. Te diffusion was relatively stable, with most Te remaining within the Bi‐Sb‐based thermoelectric material. Bi and Sb atoms diffused into the β‐Sn matrix during reflow soldering and the initial aging stage, forming a Sn(Bi, Sb) substitutional solid solution without secondary phase precipitation. Sn atoms rapidly diffused into the Bi‐Sb‐based thermoelectric material, reacting with the remaining Sb and Te to form SbSn and SnTe, creating a new Sn‐Bi‐Sb‐Te layer. As aging continued, accelerated Sn diffusion caused the Sn‐Bi‐Sb‐Te layer to thicken further, while Sb and Bi continued diffusing into the β‐Sn matrix without Bi secondary phase precipitation. Sb diffusion into β‐Sn resulted in the formation of a new SbSn secondary phase, which became an important pathway for Bi diffusion. Nanometer‐scale Bi precipitates were observed within the SbSn phase, with Bi enrichment occurring at the β‐Sn/SbSn interface. This Bi enrichment potentially caused localized eutectic melting along the β‐Sn/SbSn interface, where the Bi concentration was highest.

Figure 23b analyzes the phase composition, atomic arrangement, and growth direction of each IMC layer at the β‐Sn/Sn‐Sb/Sn‐Bi‐Sb‐Te/Bi‐Sb‐Te multi‐interface.^[^ ^272^ ^]^ The β‐Sn and SnTe phases exhibit body‐centered tetragonal (BCT) and face‐centered cubic (FCC) crystal structures, respectively, which can form solid solutions with Sb and Bi atoms, promoting their diffusion. Bi, SbSn, and Bi_0.5_Sb_1.5_Te_3_ exhibit hexagonal close‐packed (HCP) structures with similar atomic arrangements in their (001) planes. The atomic migration along the <110> direction provides a low‐energy barrier for SbSn phase growth and the movement of nanoscale Bi domains. When the concentration of dissolved Bi atoms in the β‐Sn matrix reaches a threshold (≈18 wt.%), localized eutectic melting occurs at the interface, forming a liquid phase containing Sn and Bi. During solidification of this liquid phase, the β‐Sn matrix and Sn‐Bi eutectic structures develop and distribute at the β‐Sn/SbSn interface. As more Bi atoms diffuse into β‐Sn, the β‐Sn layer thickens, ultimately reaching the conditions for localized eutectic melting. The liquid phase formed from eutectic melting can be partially extruded from the solder joint due to gravity, leading to the appearance of non‐wettable solidified cracks on the surface. The solder thickness reduces significantly, from 82 to 37 µm. This substantial reduction in thickness cannot be solely attributed to diffusion and is instead attributed to a process of localized eutectic melting, liquid‐phase extrusion, and solidification at room temperature. The study indicates that non‐wettable interfacial cracks caused by localized eutectic melting and solidification during aging are the primary failure mechanism for TED electrodes. To mitigate such failures, it is recommended to maintain the operating temperature of Bi‐Te‐based TEDs below the Sn‐Bi eutectic temperature of 139 °C and further reduce it if possible.

## 6 Challenge and Outlooks

**Figure 24:**
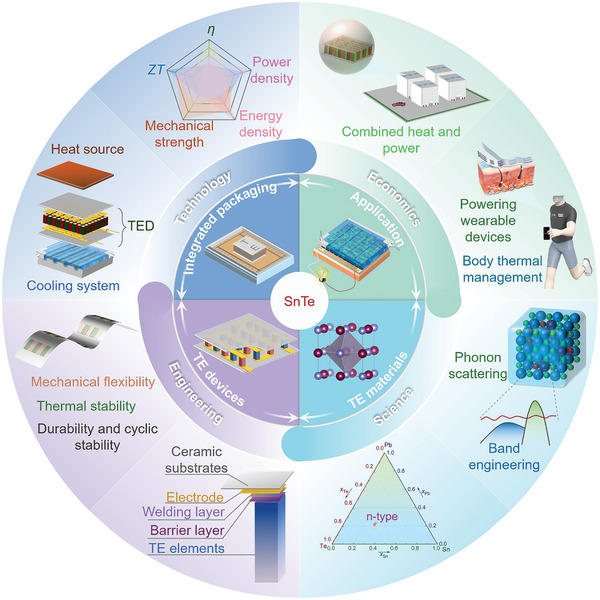
Challenges and outlooks for SnTe‐based thermoelectrics.

SnTe‐based materials have emerged as a promising class of high‐performance thermoelectric materials and have garnered extensive attention in recent years. SnTe exhibits excellent thermoelectric properties, particularly in the mid‐to‐high temperature range, with significantly enhanced *ZT* values. Through strategies such as doping, alloying, composite formation, lattice defect engineering, and 2D structure design, SnTe‐based materials have demonstrated exceptional potential in optimizing electrical transport properties and suppressing *κ*. Notably, alloying and nanostructure design have further enhanced carrier transport properties while effectively reducing *κ*_l_, opening new avenues for the advancement of SnTe‐based materials. In TEDs, SnTe‐based materials have been successfully applied in mid‐to‐high temperature power generation devices, showing great potential in waste heat recovery and energy conversion. Particularly, p‐type SnTe‐based materials have been extensively studied in TED applications. However, the poor performance of n‐type SnTe‐based materials limits the widespread application of all‐SnTe TEDs. Currently, research on SnTe‐based TEDs often relies on combining p‐type SnTe with n‐type non‐SnTe materials, leading to challenges like interface stability and mismatches in thermal expansion coefficients. Despite significant progress in the development of SnTe‐based materials and TEDs, numerous challenges remain in enhancing thermoelectric performance and achieving industrial‐scale applications, as illustrated in **Figure** **24**.

1. **n‐type SnTe**. Although p‐type SnTe‐based thermoelectric materials have made significant progress, n‐type SnTe materials remain extremely scarce, and the *ZT* values of the developed n‐type SnTe materials are still relatively low, limiting their application in high‐efficiency TEDs. To achieve high‐performance all‐SnTe‐based TEDs, the development and enhancement of n‐type SnTe materials have become the core focus of current research. Strategies such as doping, defect engineering, and crystal structure optimization hold promise for improving the performance of n‐type SnTe. However, this process faces multiple technical and theoretical challenges, requiring innovative approaches and deeper understanding to overcome existing limitations.
2. **Mature device design**. Limited by the development of n‐type SnTe thermoelectric materials, current SnTe‐based TEDs are generally assembled using p‐type SnTe materials paired with n‐type non‐SnTe materials. However, significant differences in the thermal expansion coefficients between these materials pose challenges to the mechanical and thermal stability of TEDs. Therefore, optimizing the stability of p‐type SnTe legs and developing compatible n‐type SnTe‐based legs are crucial to enhancing TED reliability. Moreover, issues related to interface design between legs, electrodes, and substrates persist, particularly due to high and unstable interface resistance, which severely affects the current transmission efficiency of TEDs. Future research should focus on optimizing interface design and structural adjustments to reduce interface resistance and improve the stability and overall performance of the devices.
3. **Device packaging**. The integration and packaging technologies for SnTe‐based TEDs are still in their early stages and face numerous challenges. Packaging TEDs involves not only encapsulating individual devices but also integrating multiple thermoelectric modules into a cohesive thermoelectric power generation system while balancing thermoelectric performance with multifunctionality, such as sensing, data acquisition, and intelligent control. Packaging technologies encompass various aspects, including thermal management (heat dissipation design, thermal interface materials), mechanical stress management (choice of packaging materials, mechanical structure design), electrical connections (electrode contacts, electrical insulation), and packaging processes (welding, bonding techniques). Different types of packaging (flexible and rigid) must address their respective mechanical stress and fatigue issues. Future packaging technologies will focus on efficient thermal management, mechanical performance, long‐term stability, and compatibility with system integration, driving the widespread adoption of TEDs in practical applications.
4. **Flexible TED**. With the advancement of smart devices and wearable technologies, research on flexible TEDs will continue to be a focal point. However, progress in applying SnTe‐based thermoelectric materials to flexible devices has been relatively slow. This is partly due to their poor flexibility and partly due to their low near‐room‐temperature *ZT* values. Enhancing the flexibility of these materials while improving their high near‐room‐temperature thermoelectric performance will be a crucial direction for future research.
5. **Broadening application scenarios**. Although SnTe‐based thermoelectric materials hold great potential for applications in thermoelectric power generation and cooling, their practical deployment remains limited due to constraints in *η*. Particularly in fields such as high‐efficiency energy recovery and industrial thermal management, the expansion of SnTe‐based TED applications faces both economic and technical challenges. Key factors driving their commercialization include reducing manufacturing costs, improving device performance, and addressing the feasibility of large‐scale applications.

In summary, SnTe‐based thermoelectric materials and devices offer significant potential for thermoelectric applications, yet face challenges in performance enhancement, device stability, integration, and packaging. The development of SnTe‐based thermoelectric technologies and products depends on breakthroughs in three fundamental areas: material preparation, device fabrication, and system integration, alongside effective thermal management solutions. With ongoing advancements in materials science, engineering technologies, and thermoelectric system integration, SnTe‐based thermoelectric devices are expected to achieve improved performance and enable groundbreaking applications in energy recovery and intelligent thermal management.

## Conflict of Interest

The authors declare no conflict of interest.
